# NAD^+^-Glycohydrolase Promotes Intracellular Survival of Group A *Streptococcus*

**DOI:** 10.1371/journal.ppat.1005468

**Published:** 2016-03-03

**Authors:** Onkar Sharma, Maghnus O’Seaghdha, Jorge J. Velarde, Michael R. Wessels

**Affiliations:** Division of Infectious Diseases, Boston Children’s Hospital, and Department of Pediatrics, Harvard Medical School, Boston, Massachusetts, United States of America; The University of Alabama at Birmingham, UNITED STATES

## Abstract

A global increase in invasive infections due to group A *Streptococcus* (*S*. *pyogenes* or GAS) has been observed since the 1980s, associated with emergence of a clonal group of strains of the M1T1 serotype. Among other virulence attributes, the M1T1 clone secretes NAD^+^-glycohydrolase (NADase). When GAS binds to epithelial cells *in vitro*, NADase is translocated into the cytosol in a process mediated by streptolysin O (SLO), and expression of these two toxins is associated with enhanced GAS intracellular survival. Because SLO is required for NADase translocation, it has been difficult to distinguish pathogenic effects of NADase from those of SLO. To resolve the effects of the two proteins, we made use of anthrax toxin as an alternative means to deliver NADase to host cells, independently of SLO. We developed a novel method for purification of enzymatically active NADase fused to an amino-terminal fragment of anthrax toxin lethal factor (LFn-NADase) that exploits the avid, reversible binding of NADase to its endogenous inhibitor. LFn-NADase was translocated across a synthetic lipid bilayer *in vitro* in the presence of anthrax toxin protective antigen in a pH-dependent manner. Exposure of human oropharyngeal keratinocytes to LFn-NADase in the presence of protective antigen resulted in cytosolic delivery of NADase activity, inhibition of protein synthesis, and cell death, whereas a similar construct of an enzymatically inactive point mutant had no effect. Anthrax toxin-mediated delivery of NADase in an amount comparable to that observed during *in vitro* infection with live GAS rescued the defective intracellular survival of NADase-deficient GAS and increased the survival of SLO-deficient GAS. Confocal microscopy demonstrated that delivery of LFn-NADase prevented intracellular trafficking of NADase-deficient GAS to lysosomes. We conclude that NADase mediates cytotoxicity and promotes intracellular survival of GAS in host cells.

## Author Summary

Invasive infections due to group A *Streptococcus* (*S*. *pyogenes* or GAS) have become more frequent since the 1980s due, in part, to the emergence and global spread of closely related strains of the M1T1 serotype. A feature of this clonal group is the production of a secreted enzyme, NAD^+^-glycohydrolase (NADase), which has been suggested to contribute to GAS virulence by intoxication of host cells. For NADase to exert its toxic effects, it must be translocated into the host cell by a second GAS protein, streptolysin O (SLO). SLO is a pore-forming toxin that damages cell membranes in addition to its role in translocating NADase. In order to distinguish effects of NADase on host cell biology from those of SLO, we used components of anthrax toxin to deliver NADase to human throat epithelial cells, independently of SLO. Introduction of NADase into GAS-infected cells increased the intracellular survival of GAS lacking NADase or SLO, and the increase in bacterial survival correlated with inhibition of intracellular trafficking of GAS to lysosomes that mediate bacterial killing. The results support an important role for NADase in enhancing GAS survival in human epithelial cells, a phenomenon that may contribute to GAS colonization and disease.

## Introduction

Since the 1980’s, there has been a sustained, worldwide increase in the incidence of severe, invasive infections due to group A *Streptococcus* (*Streptococcus pyogenes* or GAS), particularly necrotizing fasciitis and streptococcal toxic shock syndrome [1–3]. The reasons for the emergence of invasive GAS disease are incompletely understood; however, a partial explanation may be the global dissemination of a clonal group of strains of the M1T1 serotype. The invasive M1T1 strains harbor bacteriophage-associated genes encoding such virulence factors as the pyrogenic exotoxin SpeA and the secreted DNase Sda1 (also called SdaD2), both of which have been associated with GAS pathogenicity in model systems. In addition, these strains secrete NAD^+^-glycohydrolase (NADase), a property that generally was not present among M1 strains isolated prior to 1988 [4–6]. NADase is encoded by *nga*, which is located in an operon together with *ifs*, encoding an intracellular inhibitor that dissociates from NADase upon NADase secretion, and *slo* encoding the cholesterol-dependent cytolysin/hemolysin, streptolysin O (SLO) [4,7–9]. Genomic analyses of multiple M1 isolates from the past century indicate that the invasive M1T1 strain acquired a 36-kb chromosomal region that includes the *nga* and *slo* genes prior to emergence of this strain in the 1980s [10–12]. The association of NADase activity with contemporary invasive M1T1 isolates has suggested that production of the enzyme might contribute to virulence.

Physical association of NADase with hemolytic activity in GAS culture supernatants led to early misidentification of NADase and SLO as a single protein, although subsequent studies clearly separated the two [13–15]. A new paradigm for the interaction of NADase and SLO was proposed by Madden *et al*., who found that NADase could be translocated into the cytosol of epithelial cells *in vitro* after its secretion from GAS bound to the cell surface [16]. Translocation required the concomitant expression of SLO, which suggested a model in which NADase associates with SLO on the epithelial cell surface and is transferred across the cell membrane in a process dependent on SLO. These and subsequent studies provided evidence that SLO-mediated delivery of NADase augmented the cytotoxic effect of SLO and induced epithelial cell apoptosis [16,17]. NADase-deficient mutants were found to have reduced virulence in mice compared to wild type GAS, supporting a role of the enzyme in pathogenesis of invasive infection [18,19].

The exposure of human oropharyngeal keratinocytes to GAS that produce both SLO and NADase, but not to those producing SLO alone, results in depletion of intracellular NAD^+^ and ATP. This finding is consistent with the enzymatic function of NADase to hydrolyze cellular NAD^+^ to nicotinamide and adenosine diphosphoribose and, secondarily, to deplete cellular ATP [20]. In previously published work, we used isogenic mutants deficient in SLO or NADase to study the role of each toxin in enhancing intracellular survival of GAS. These studies revealed that NADase-deficient GAS are more efficiently killed after internalization by keratinocytes compared to SLO^+^NADase^+^ GAS [21]. The increased survival of NADase-producing strains is associated with failure of GAS-containing vacuoles to fuse with lysosomes to form an acidic, bactericidal compartment [21]. While these observations have suggested a role for NADase in GAS pathogenesis, prior studies have been limited in their capacity to distinguish effects of NADase from those of SLO, since SLO is itself a cytotoxin and is required to deliver NADase to host cells. The goal of the present investigation was to distinguish effects of NADase from those of SLO during interaction of GAS with human epithelial cells. To this end, we developed a system that delivers NADase to the cytosol of host cells independently of SLO. Utilizing the anthrax toxin platform to deliver enzymatically active or inactive forms of recombinant NADase to cells infected with various GAS strains, we have obtained direct evidence that the catalytic activity of NADase is a critical effector of GAS intracellular trafficking and intracellular survival.

Anthrax toxin, the major virulence factor of *Bacillus anthracis*, is an A-B type toxin composed of the catalytic moieties, lethal factor (LF) and edema factor (EF), and the receptor binding/pore forming protective antigen (PA; MW 83 kDa). Upon release by the bacteria, PA_83_ binds to its cellular receptors and is cleaved by cell surface furin to a 63 kDa form (PA_63_), which then self-assembles to form a heptameric or octameric prepore [22–24]. The prepore binds the enzymatic LF and/or EF moieties to form complexes that are subsequently endocytosed [25,26]. The low pH of the endosome causes the PA prepore to undergo a conformational change into the pore form, which inserts into the endosomal membrane and translocates the catalytic LF and EF moieties into the cytoplasm [27–29].

The intrinsic activity of the anthrax toxin system for intracellular delivery of its catalytic components can be harnessed to translocate heterologous proteins into the cytosol of its target cells. Fusing the non-catalytic N-terminal PA-binding domain of LF (LFn, residues 1 to 263)) [30] to any of a variety of unrelated “cargo” proteins enables them to undergo PA-dependent translocation to the cytosol. Examples include a cytotoxic T lymphocyte epitope from *Listeria monocytogenes*, the gp120 portion of the HIV-1 envelope protein, and the activity domains of *Pseudomonas* exotoxin, diphtheria toxin, or shiga toxin [31–35]. In the current study, we fused LFn to NADase or its variants and utilized the anthrax toxin platform to deliver enzymatically active or inactive forms of the enzyme to human oropharyngeal keratinocytes independently of SLO. Results of *in vitro* infection experiments utilizing this system provide direct evidence that the enzymatic activity of NADase is a critical effector of GAS intracellular trafficking and survival.

## Results

### A novel expression and purification protocol yields enzymatically active NADase alone and fused to anthrax toxin lethal factor N-terminal domain (LFn)

Functional analysis of GAS NADase has been complicated by the necessity to co-express its endogenous inhibitor IFS (Immunity Factor for Streptococcal NADase) to prevent toxicity to the cell that produces the active enzyme [8,9]. IFS must be removed for NADase to be enzymatically active. Previously, expression and purification of NADase in *E*. *coli* was achieved by directing secretion of recombinant NADase to the periplasmic space, allowing IFS to remain in the cytosol [9,36]. In our hands, the yield was low with this approach, and a portion of IFS remained in the NADase-containing fraction, presumably due to incomplete exclusion of cytosolic proteins in the periplasmic preparation.

**Fig 1:**
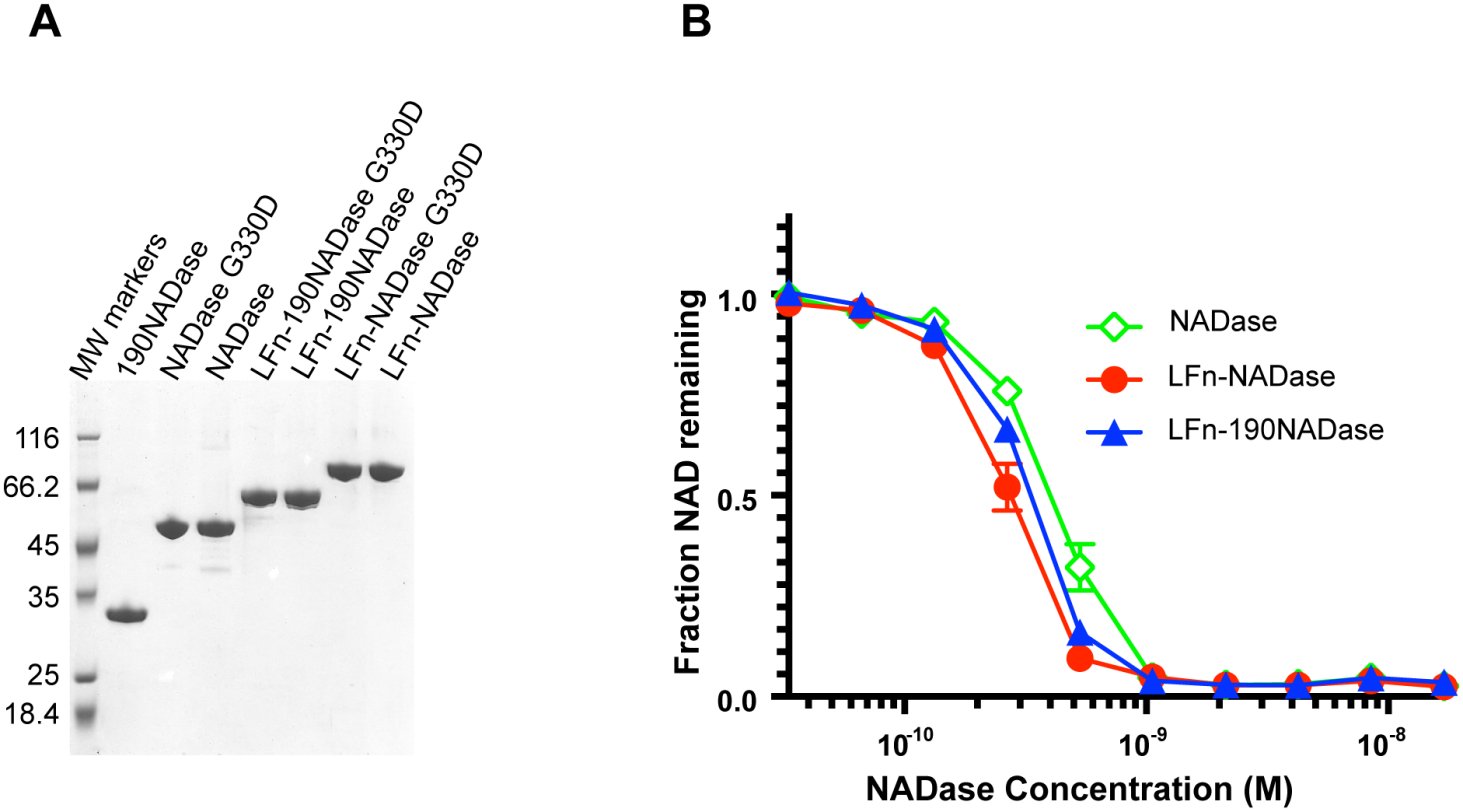
Purification and biochemical characterization of recombinant proteins. (A) Coomassie-stained SDS-PAGE of purified recombinant proteins. Proteins LFn-NADase, LFn-190NADase, NADase, and 190NADase were released from IFS and purified using a novel protocol in which the proteins were denatured, purified, and then refolded (Materials and Methods). (B) Recombinant LFn-NADase, LFn-190NADase, and NADase proteins had similar NADase activities after renaturation.

In order to produce sufficient quantities of NADase free of IFS, we developed a novel scheme for expression and purification. Because initial experiments indicated low expression levels of NADase and its fusion constructs, the *nga* and *ifs* gene sequences were codon-optimized for expression in *E*. *coli*. We then exploited the high-affinity binding of IFS to NADase to purify native and variant forms of the enzyme and various fusion constructs using His_6_-tagged IFS (S1 Fig). In the first step, we purified the NADase-IFS-His_6_ complex, which bound to a Ni-charged resin. His_6_-tagged IFS was then released from untagged NADase by denaturing the two proteins with guanidinium chloride. A second round of affinity chromatography was used to separate His_6_-tagged IFS, which was retained by the Ni column, from untagged NADase in the flow through fraction. NADase was then refolded slowly by removal of guanidinium chloride by dialysis. High protein purity was achieved by Q column purification of proteins after the first Ni column affinity purification, and then again after renaturation of IFS-free NADase constructs. Each of the purified recombinant proteins migrated predominantly as a single band of the expected molecular size on SDS-PAGE (Fig 1A).

In addition to native NADase, two variant forms were expressed and purified, both as individual proteins and as fusions to LFn. Variant 190NADase lacks the N-terminal 190 amino acids required for SLO-dependent translocation of NADase [37]; NADaseG330D harbors a point mutation that almost completely abrogates NAD-glycohydrolase activity [6,38,39]. Since the protocol involved protein denaturation and renaturation, we confirmed that the purified LFn-NADase, LFn-190NADase, and NADase proteins retained similar levels of NAD-glycohydrolase activity (Fig 1B). The K_cat_ value for LFn-NADase was estimated at 4200 reactions/sec, which compares favorably with published estimates of 3700 and 8000 reactions/sec, determined for purified NADase using a highly sensitive HPLC-based assay [36,38]. LFn-NADaseG330D and NADaseG330D lacked detectable catalytic activity. However, both LFn-NADaseG330D and NADaseG330D were able to compete with NADase for binding of IFS after renaturation (S2 Fig). We also analyzed the secondary structure of purified recombinant NADaseG330D by circular dichroism spectroscopy and found nearly identical results as those for purified recombinant (and enzymatically active) NADase (S2 Fig). Together, these analyses provide evidence that renaturation of the enzymatically inactive variants LFn-NADaseG330D and NADaseG330D restored the native conformations of the purified proteins.

### LFn-NADase is translocated across PA pores in planar lipid bilayers

**Fig 2:**
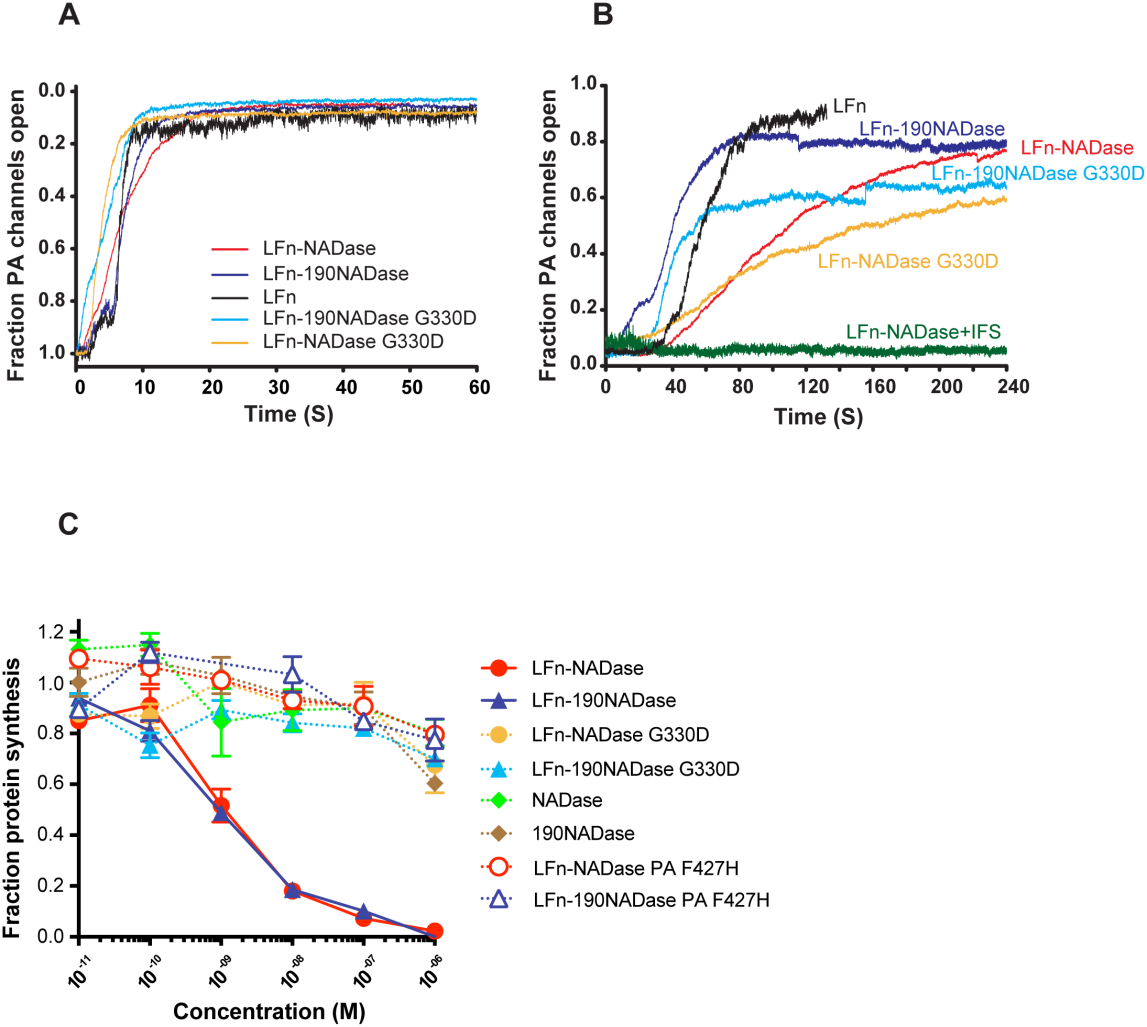
Anthrax toxin-mediated translocation of NADase. (A) Kinetics of occlusion of PA pores in planar bilayers by LFn-NADase, LFn-190NADase, LFnNADase G330D, and LFn-190NADase G330D. Interaction with PA channels was monitored by the decrease in conductance, which is plotted as fraction of open PA channels. (B) Translocation of LFn-NADase, LFn-190NADase, LFn-NADase G330D and LFn-190NADase G330D proteins across PA channels in planar bilayers. After complete occlusion of PA channels, KOH was added to the *trans* compartment raising the pH from 5.5 to 7.5, triggering translocation, which is visualized by an increase in conductance and is plotted as fraction of open PA channels. (C) Inhibition of protein synthesis by PA-mediated translocation of LFn-NADase or variants in OKP7 cells. Protein synthesis was assayed by incorporation of tritiated leucine and is plotted as the fraction of activity in OKP7 cells in the absence of recombinant protein.

We tested the ability of LFn-NADase and its variants to interact with and translocate across PA pores in planar bilayers *in vitro* as measured by ion conductance. Occlusion of pores in DPhPC bilayers was monitored for 60 sec following addition of each recombinant protein (final concentration 1 μg/ml) to the *cis* compartment. All of the constructs tested (LFn, LFn-NADase, LFn-190NADase, LFn-NADaseG330D, and LFn-190NADaseG330D) blocked conductance rapidly (within 20 sec) and almost completely (Fig 2A). Subsequently, translocation was initiated by addition of KOH to the *trans* compartment to increase the pH to ~7.5. Translocation of free LFn and LFn-190NADase, as measured by return of ion conductance, was rapid (within ~80 sec) and essentially complete (~80–90%) (Fig 2B). LFn-NADase took longer (240 sec) to achieve comparable translocation. LFn-NADaseG330D and LFn-190NADaseG330D constructs were less efficiently translocated, with about ~60% translocation achieved in 240 sec. Interestingly, addition of IFS to the *cis* compartment (final concentration 6 μg/ml) before addition of KOH to the *trans* compartment prevented translocation of LFn-NADase (Fig 2B). To test whether the binding of NADase to IFS prevents NADase unfolding, which is necessary for translocation, we used differential scanning fluorimetry to measure the melting temperature of NADase, IFS, and NADase-IFS complex. The melting temperatures were determined to be 43°C, 60°C and 76°C, respectively (S3 Fig). Thus, the tight binding of IFS increases the T_m_ of NADase by more than 30°C, presumably preventing its unfolding, a required step for the translocation of LFn-NADase across PA pores.

### Delivery of NADase to the cytosol of human oropharyngeal keratinocytes independently of SLO

Having determined that LFn-NADase could be translocated through PA pores in an artificial membrane *in vitro*, we investigated whether PA pores could mediate delivery of LFn-NADase into human oropharyngeal keratinocytes. We reasoned that NAD^+^-glycohydrolase activity of LFn-NADase would deplete cellular energy stores resulting in inhibition of protein synthesis. Accordingly, NADase and its variants were tested for PA-mediated translocation into OKP7 cells by measuring inhibition of cellular protein synthesis. In the presence of PA, LFn-NADase and LFn-190NADase were efficiently translocated, with half-maximal inhibition of protein synthesis observed at a LFn-NADase concentration of ~1 nM in the cell culture medium (Fig 2C). LFn-190NADase gave an almost identical result, a finding that implies the N-terminal domain of NADase involved in SLO-mediated translocation is dispensable for delivery of the enzyme by the anthrax toxin system. In the absence of PA, no LFn-NADase translocation was observed (S4 Fig), and, as expected, NADase and 190NADase also did not translocate. Translocation of the enzymatically inactive forms of NADase, LFn-NADaseG330D and LFn-190NADaseG330D, did not inhibit cellular protein synthesis, even at concentrations up to 1,000 times that required for inhibition by LFn-NADase (Fig 2C).

A key determinant of translocation by PA is the phenylalanine clamp, a structure formed by the F427 side chains within the lumen of the PA pore [40]. We tested LFn-NADase and LFn-190NADase for cytotoxicity in the presence of PA F427H, a mutant form of PA, which forms pores that lack the ability to mediate translocation. The F427H mutation completely blocked LFn-NADase translocation (Fig 2C), implying PA-mediated translocation is dependent on interaction with the Phe clamp and occurs through the central pore.

### NADase-mediated cytotoxicity is due to NAD^+^-glycohydrolase activity

**Fig 3:**
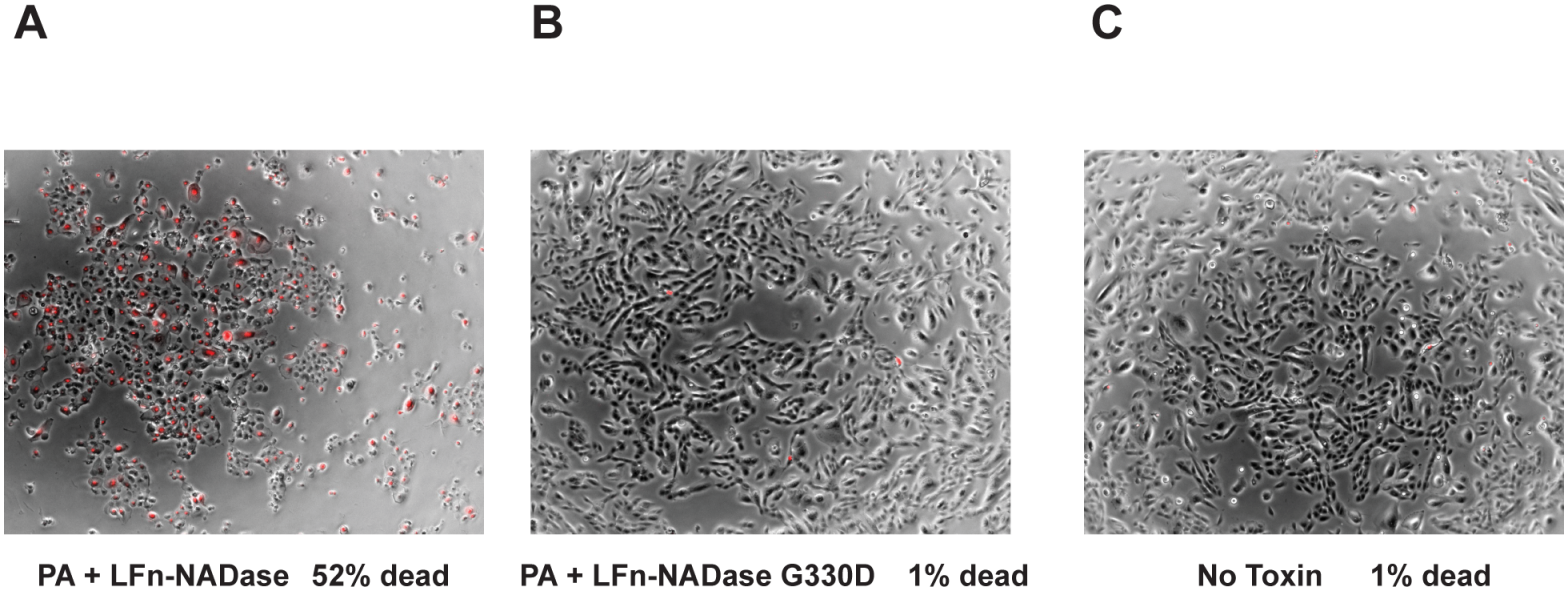
Cytotoxic effects of NADase depend on enzymatic activity. (A) Propidium iodide uptake (red) indicating cell membrane damage is superimposed on bright field images of OKP7 cells exposed to LFn-NADase or (B) enzymatically inactive LFn-NADase G330D, both in the presence of anthrax toxin PA, or (C) untreated control cells.

It has been suggested that introduction of NADase into host cells exerts cytotoxic effects that are independent of NAD^+^-glycohydrolase activity of the protein [38]. The anthrax toxin delivery system enabled us to test this hypothesis in the absence of other GAS virulence factors. We found that exposure of OKP7 keratinocytes to LFn-NADase in the presence of PA resulted in rounding, pyknosis, and uptake of propidium iodide indicating loss of cell viability (Fig 3). Treatment with LFn-NADase resulted in 52% cell death as assessed by propidium iodide staining. In addition, treatment with LFn-NADase caused significant cell loss when compared to untreated cells, presumably due to cells becoming non-adherent upon loss of viability. In contrast, identical exposure to enzymatically inactive LFn-NADaseG330D in the presence of PA caused no cytotoxicity compared to untreated cells (1% cell death for each condition). These results provide direct evidence that the cytotoxic effects of NADase are due solely to its enzymatic activity.

### PA-mediated translocation delivers LFn-NADase in an amount comparable to that associated with exposure of cells to live GAS

**Fig 4:**
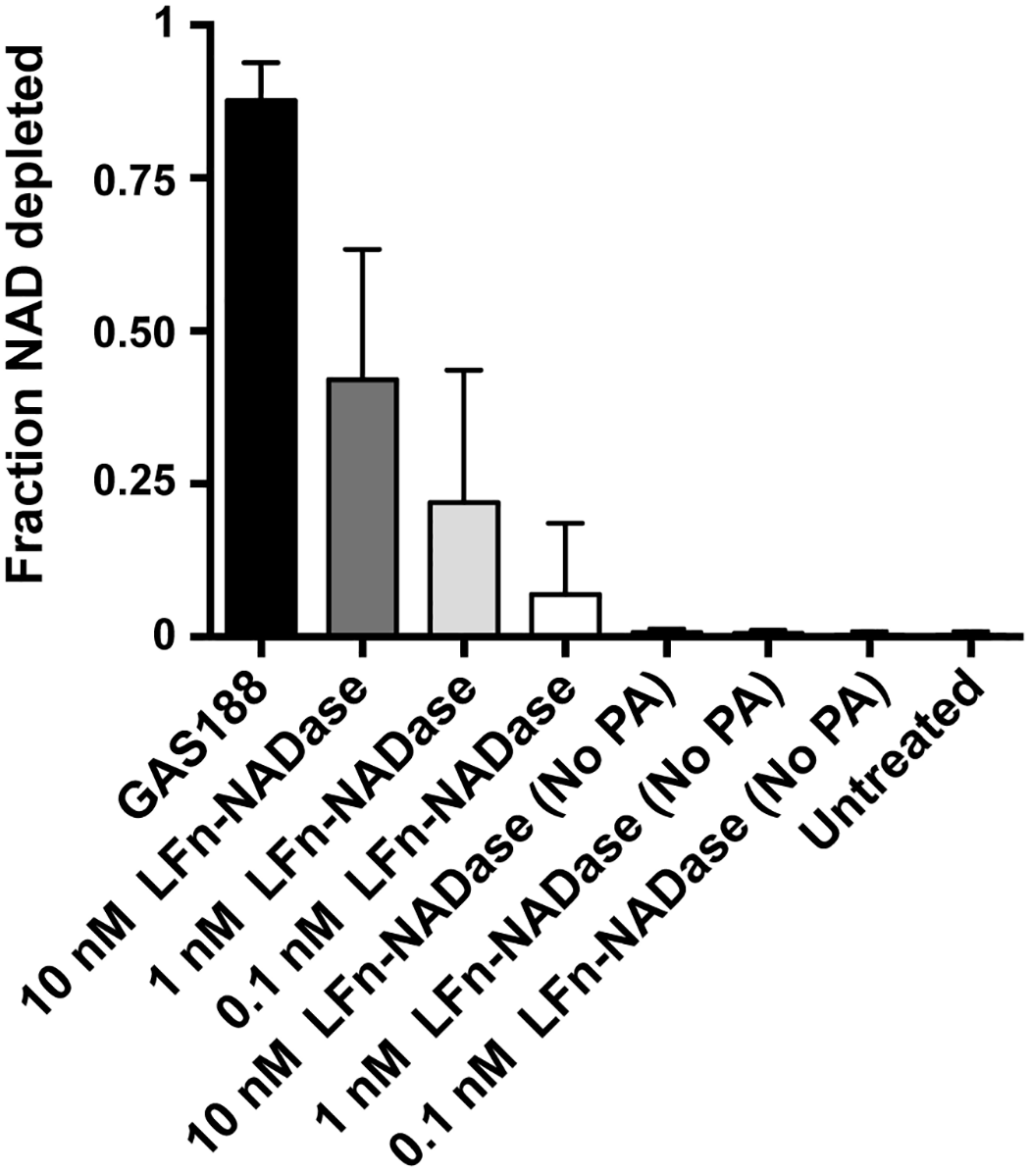
Intracellular NADase activity during infection by GAS compared with that delivered by LFn-NADase. Cells were exposed to GAS strain 188 or to anthrax toxin PA and LFn-NADase for 2 h, after which cytosolic NADase activity was measured. Data represent the fraction of NAD^+^ substrate depleted by the cytosolic fraction of treated cells. Experiments were performed 3 times, and results are shown as mean±SEM.

Previous studies on the effects of NADase on epithelial cells have utilized model systems in which cells are exposed to live GAS *in vitro* [17,21]. In order to compare effects of NADase delivered by the anthrax toxin system with those associated with exposure to live GAS, we measured intracellular NAD-glycohydrolase activity under both conditions. Our goal was to determine the concentration of LFn-NADase to be added to OKP7 cells so that the subsequent PA-mediated delivery would result in an intracellular NADase activity comparable to that achieved by exposure to live GAS in prior studies. We found that addition of LFn-NADase to a concentration of 10 nM achieved a level of NADase activity in the cytosol of the keratinocytes that corresponded to approximately 50% of that associated with infection by NADase-producing GAS strain 188 at an MOI of 10 (Fig 4, S5 Fig).

### Enzymatically active NADase rescues the defective intracellular survival of NADase-deficient GAS

**Fig 5:**
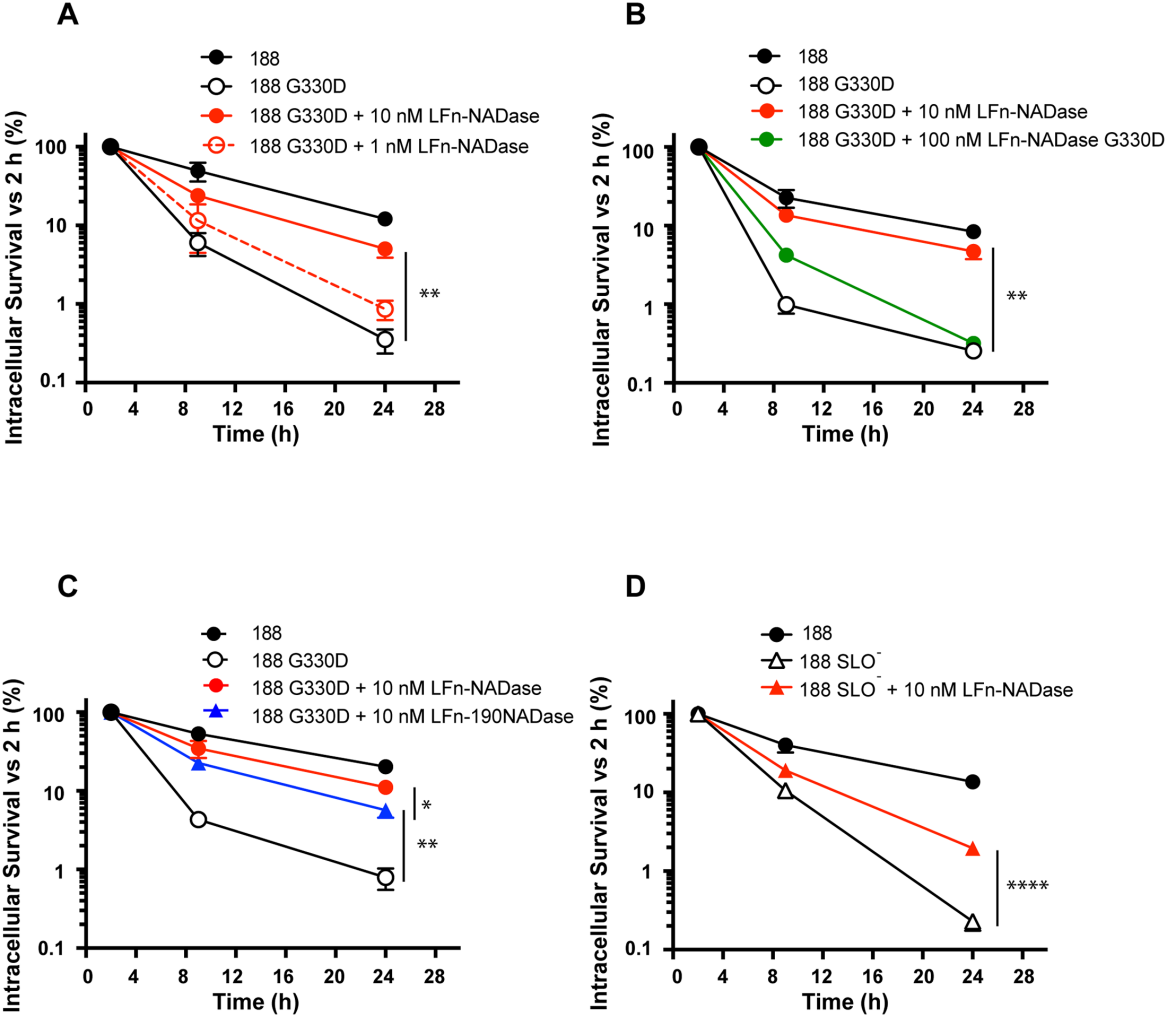
Effect of anthrax toxin-mediated delivery of LFn-NADase or variants on the intracellular survival of GAS deficient in enzymatically active NADase (188 G330D) or SLO (188 SLO^-^). Survival of intracellular GAS at the indicated time points is plotted as percent of CFU recovered at 2 hours of infection. (A) Dose-dependent increase in survival of 188 G330D in the presence of LFn-NADase. (B) Increase in survival of 188 G330D in the presence of LFn-NADase but not in the presence of enzymatically inactive LFn-NADase G330D. (C) Increase in survival of 188 G330D in the presence of either LFn-NADase or LFn-190NADase, which lacks the N-terminal translocation domain. (D) Increase in survival of 188 SLO^-^ in the presence of LFn-NADase. Values represent mean±SEM of at least 3 independent experiments. *, *P*<0.05; **, *P*<0.01; ****, *P*<0.0001.

Infection of OKP7 cells with GAS is associated with survival of 10 to 15% of intracellular bacteria, whereas fewer than 1% of GAS deficient in NADase activity survive intracellularly for 24 hours [21]. We reasoned that anthrax toxin-mediated delivery of exogenous NADase might rescue intracellular GAS that did not produce enzymatically active NADase. We found that addition of 10 nM LFn-NADase to OKP7 cells in the presence of 20 nM PA increased the intracellular survival of GAS strain 188 G330D, which expresses enzymatically inactive NADase, by 14-fold, from 0.35% at 24 hours to 5% (Fig 5A). Thus, addition of exogenous NADase restored intracellular GAS survival to an extent roughly commensurate with the amount of NADase activity delivered to the cytosol of the host cell, i.e., approximately 50% of the activity associated with infection of the cell by the parent strain, 188. Addition of 1 nM LFn-NADase had a lesser and not statistically significant effect, increasing survival at 24 h of 188 G330D 2.5-fold to 0.9%. The ability of exogenously delivered NADase to restore intracellular GAS survival was dependent on the catalytic activity of the protein: addition of LFn-NADaseG330D had no effect on the 24-hour survival of GAS within OKP7 cells, even at 100 nM (Fig 5B).

The process of SLO-dependent translocation of NADase across the eukaryotic cell membrane requires a 190-aa domain in the amino terminus of the NADase protein, a part of the molecule that is dispensable for enzymatic activity [37]. As suggested by the protein synthesis inhibition assays (Fig 2C), we found that LFn-190NADase could function in lieu of LFn-NADase to increase the intracellular survival of 188 G330D, albeit slightly less efficiently than LFn-NADase (7-fold increase in survival versus 14-fold for LFn-NADase, Fig 5C). These results imply that the catalytic domain of NADase plays a dominant role in the intracellular survival of GAS. However, the small but reproducible improvement in survival imparted by LFn-NADase compared to LFn-190NADase suggests that the N-terminal translocation domain has an as-yet-unidentified function in enhancing intracellular survival.

### Intracellular delivery of NADase enhances survival of SLO-deficient GAS

The anthrax toxin delivery system allowed us to evaluate the contribution of NADase to GAS intracellular survival in the absence of SLO. Because SLO is ordinarily required to translocate NADase during GAS infection, it has not been possible previously to assess the role of NADase on GAS intracellular survival independently of SLO. To address the discrete contribution of each toxin, we added LFn-NADase and PA during infection of OKP7 cells with 188 SLO^-^ and assessed the effect on intracellular survival (Fig 5D). Delivery of LFn-NADase increased intracellular survival of 188 SLO- by ~8 fold, from 0.25% to 2.0%. Thus, delivery of NADase prolongs the survival of both 188 G330D and 188 SLO^-^ strains, results that imply both SLO and NADase are required for maximum resistance to intracellular killing. The fact that SLO-independent delivery of NADase partially corrects the survival defect of an SLO-deficient strain indicates that the reduced survival of SLO- GAS is due in part to the absence of NADase delivery, but also that SLO possesses NADase-independent activities that contribute to the intracellular survival of GAS. Thus, the synergistic action of SLO and NADase mediates optimal intracellular survival.

### NADase prevents trafficking of intracellular GAS to lysosomes for efficient killing

**Fig 6:**
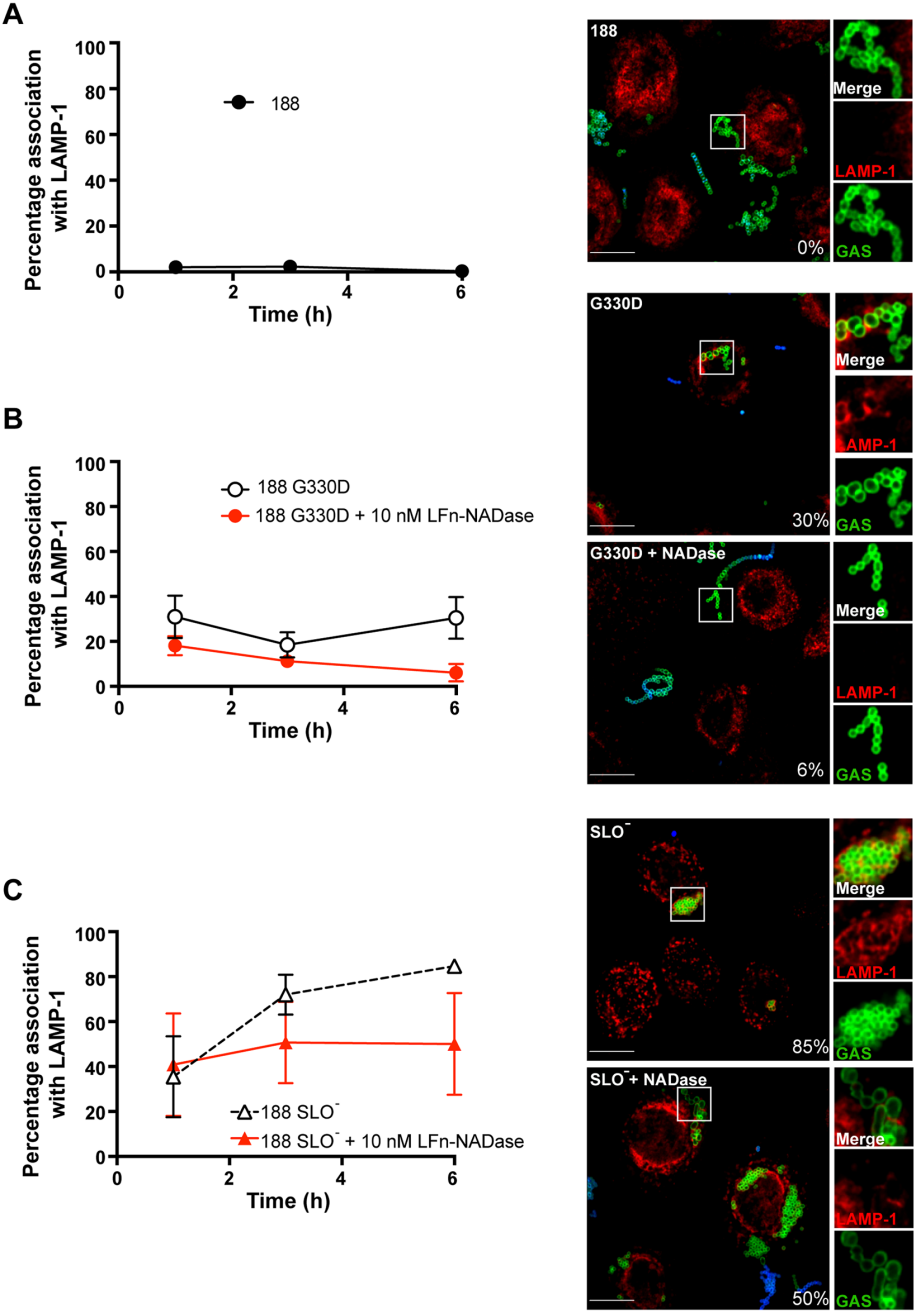
Effect of anthrax toxin-mediated delivery of LFn-NADase on intracellular trafficking of GAS. LFn-NADase inhibits trafficking of GAS deficient in expression of enzymatically active NADase (188 G330D) or SLO (188 SLO^-^) to lysosomes. (Left panels) Data points indicate percent co-localization of intracellular GAS with the lysosomal protein LAMP-1 as assessed by confocal microscopy at 1, 3, or 6 hours of infection of OKP7 cells in the absence or presence of LFn-NADase. Values represent mean±SEM of at least 3 independent experiments. (Right panels) Representative confocal microscopy images at 6 hours of infection. Intracellular GAS are labeled green (Alexa-488), extracellular GAS are labeled blue (Alexa-660) and green, and LAMP-1 is labeled red (Alexa-568). Percent co-localization of GAS with LAMP-1 is indicated. Scale bar, 10 μm. (A) Infection with GAS strain 188; (B) Infection with GAS strain 188 G330D alone or with PA-mediated delivery of LFn-NADase; (C) Infection with GAS strain 188 SLO- alone or with PA-mediated delivery of LFn-NADase. *, *P*<0.05; ***, *P*<0.001.

Previous studies have implicated SLO and NADase in GAS resistance to killing by epithelial cells. After internalization, SLO-deficient mutants are contained within endosomes or autophagosomes that fuse with lysosomes, an event associated with acidification of the GAS-containing vacuole and efficient bacterial killing [21,41]. The anthrax toxin system allowed us to assess directly the ability of NADase to interfere with fusion of the GAS-containing compartment with lysosomes. We found that delivery of exogenous LFn-NADase to the cytosol of GAS-infected OKP7 cells reduced the co-localization of 188 G330D with the lysosomal marker LAMP-1 (Lysosomal–Associated Membrane Protein 1) by 4-fold, from 41% to 10% at 6 h of infection (*P*<0.001, Fig 6). Delivery of LFn-NADase also inhibited trafficking of 188 SLO^-^ to a LAMP-1-positive compartment, reducing co-localization with LAMP-1 from 86% to 51% (*P*<0.05). These findings correlate with the effects of LFn-NADase on the intracellular survival of 188 G330D and 188 SLO^-^ and provide direct evidence that NADase contributes to GAS intracellular survival by interfering with lysosomal fusion to the GAS-containing vacuole.

**Fig 7:**
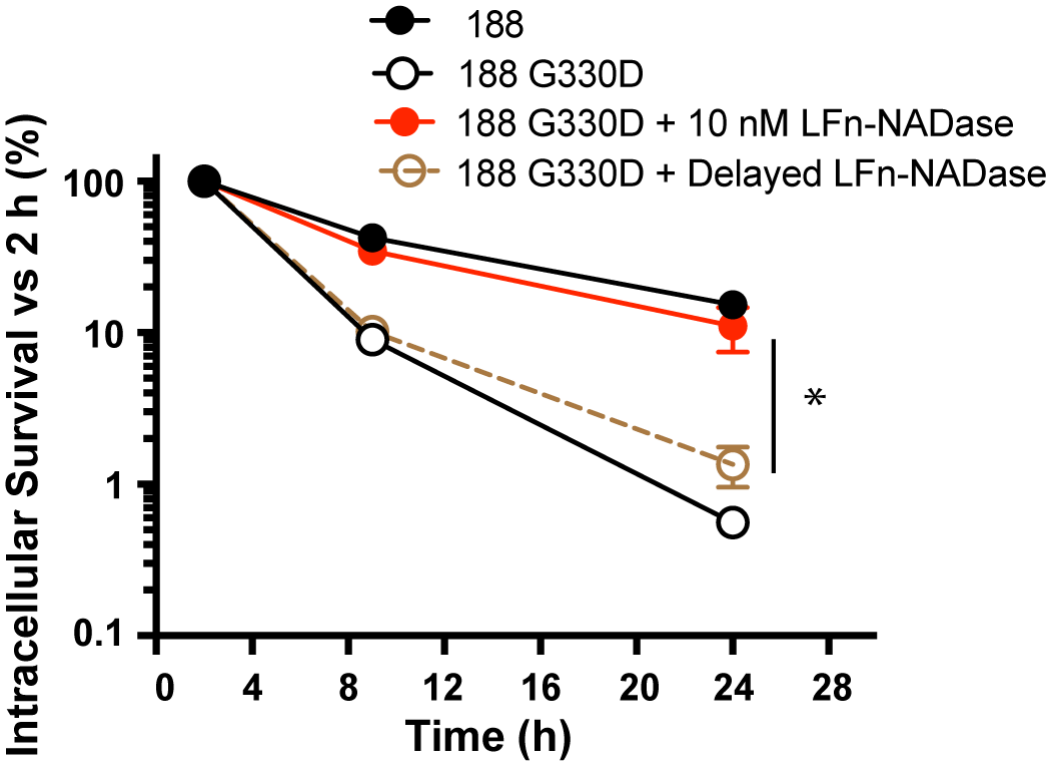
Intracellular survival of GAS 188 G330D following anthrax toxin-mediated delivery of LFn-NADase at time 0h or at 2h of infection. Values represent mean±SEM of at least 3 independent experiments. *, *P*<0.05.

Because the effect of LFn-NADase on endosomal trafficking was evident within the first few hours of infection, it seemed likely that the survival of intracellular GAS was largely determined during this time period. We found that a delay of only 2 hours in the addition of LFn-NADase to cells infected with 188 G330D largely abrogated the 24-hour survival benefit of LFn-NADase compared with that conferred by addition of the toxin at the time of initial infection (Fig 7). This result is consistent with the finding that, in the absence of NADase expression, GAS are trafficked to a degradative compartment by lysosomal fusion as early as 1 hour after infection (Fig 6).

## Discussion

A role for NADase in the virulence of GAS was suggested by the association of NADase production with M1T1 GAS isolates from invasive infections, beginning in the 1980s. Subsequent studies by Caparon and coworkers established a compelling model for SLO-dependent translocation of NADase into host cells, and intoxication of the cells was shown by our group to result in depletion of cellular NAD^+^ and ATP [16,20]. Experiments with NADase-deficient mutants supported a role for NADase in synergistic cytotoxicity with SLO, in induction of apoptosis, and in enhancing intracellular survival of GAS internalized by epithelial cells [17,38]. However, these functions of NADase during GAS infection have been inferred almost entirely from comparisons with mutants that lacked NADase or produced an enzymatically defective protein. The requirement of SLO for translocation of NADase has made it difficult to analyze the biological effects of NADase separately from those of SLO, which is required for NADase delivery, but which also has intrinsic cytotoxicity due to its pore-forming activity. An additional level of experimental complexity arises from the tightly bound endogenous inhibitor of NADase, IFS, whose co-expression is required for NADase production, but which must be removed to restore enzymatic activity.

In the current study, the anthrax toxin system provided a tractable platform to deliver enzymatically active, highly purified, IFS-free NADase or variant forms to the cytosol of human oropharyngeal keratinocytes. This system permitted direct investigation of the function of NADase in the cell biology of GAS infection, independent of the effects of SLO. We found that SLO-independent cytosolic delivery of LFn-NADase inhibited protein synthesis in oropharyngeal keratinocytes in a dose-dependent manner (Fig 2C). Nearly identical inhibition was observed upon delivery of LFn-190NADase, which lacks the N-terminal domain of NADase required for SLO-mediated translocation, but preserves the catalytic domain. By contrast, LFn-NADase G330D, an enzymatically inactive variant, had no inhibitory effect, even at high doses. Consistent with these results, sufficient doses of NADase delivered by the anthrax toxin system resulted in cytotoxicity and cell death that was dependent on the catalytic activity of the protein (Fig 3). These results support the view that the intrinsic cytotoxic activity of NADase on eukaryotic cells depends on the enzymatic activity of the toxin. Depletion of cellular NAD^+^ and ATP is expected to have a broad range of inhibitory effects on cellular functions. It remains possible that the synergistic toxicity of NADase with SLO also involves a second, non-enzymatic mechanism, as suggested by Chandrasekaran *et al*, although the molecular basis for such an effect has not been determined [38].

Previous studies found that SLO was required for prolonged GAS intracellular survival in keratinocytes [21,41]. Shortly after bacterial internalization, GAS production of SLO results in damage to the endosomal membrane, which exposes the bacteria to the cytosol where they become ubiquitinated. Ubiquitin is a signal for targeting intra-cytosolic bacteria to autophagosome-like compartments [21,42]. Fusion of lysosomes with these compartments leads to their maturation into degradative autolysosomes and efficient bacterial killing. Autophagosomes containing NADase-deficient GAS appear to follow this pathway; however, the step of lysosomal fusion is impaired for autophagosomes containing NADase-producing GAS, and this impairment is associated with enhanced intracellular survival [21]. The anthrax toxin system allowed us to study directly whether NADase prevents lysosomal fusion with GAS-containing vacuoles in infected cells. We found that cytosolic delivery of NADase inhibited the co-localization of GAS 188 G330D (expressing an enzymatically inactive NADase) with the lysosomal marker LAMP-1 (Fig 6). Inhibition of lysosomal fusion was associated with a 14-fold increase in intracellular survival to a level approaching that of the NADase-producing parent strain. Delivery of enzymatically inactive NADase G330D had no effect on GAS intracellular survival, supporting the essential role of enzymatic activity in enhancing intracellular survival. In similar experiments, we tested the effect of NADase delivery on the intracellular survival of the SLO-deficient GAS strain 188 SLO-. Supplying exogenous NADase partially rescued survival of 188 SLO-, a result that implies that SLO contributes to GAS intracellular survival in part through delivery of NADase, but also through function(s), such as pore-formation, independent of NADase translocation. These data are consistent with the observation that a GAS strain producing a non-pore-forming SLO that is competent for NADase translocation (SLO Y2552A) was defective for intracellular survival [21].

Results of these experiments provide the most direct evidence to date on the contribution of NADase to the cell biology of GAS infection. Use of the anthrax toxin delivery system isolated the effects of NADase from those of SLO and defined an unambiguous role for NADase in cytotoxicity for host epithelial cells and in enhancing GAS intracellular survival. Both functions were dependent on cytosolic delivery of NADase and on the enzymatic activity of the toxin to degrade NAD^+^. Together, these findings provide a plausible molecular basis for the association of NADase expression with GAS virulence.

## Materials and Methods

### Cell culture

The OKP7/bmi1/TERT (OKP7) keratinocytes used in this study are immortalized normal human soft palate keratinocytes [43,44]. These cells were a gift of James Rheinwald and were provided through the Harvard Skin Disease Research Center. OKP7 cells were cultured in keratinocyte serum-free medium (KSFM, Gibco/Invitrogen) as described previously [41].

### Bacterial strains and culture conditions

GAS strain 188 and its mutant derivatives were used in this study. GAS strain 188 is an isogenic unencapsulated mutant of the M type 3 necrotizing fasciitis isolate 950771 [45]. Use of an unencapsulated mutant allowed efficient internalization of GAS by human cells *in vitro* because the hyaluronic acid capsule inhibits GAS internalization. *Escherichia coli* XL1-Blue was used as a host for molecular cloning (NEB) and was grown in Luria-Bertani (LB) medium (Novagen). GAS was grown in L3 medium as described with two modifications: the final CaCl_2_ concentration was 0.015% and type 1-S bovine hyaluronidase was omitted [46].

### Cloning and mutagenesis

*Generation of LFn-NADase-IFS constructs*. The LFn-NADase-IFS-encoding construct was created by first PCR-amplifying separately the LFn-encoding sequence [47] and the *nga-ifs* genes from GAS genomic DNA. These amplicons were then used as templates for overlap PCR to generate the LFn-NADase-IFS-encoding DNA fragment, incorporating a BamHI restriction site inserted between the LFn-encoding sequence and *nga-ifs*. This product was subsequently cloned into pET43.1a vector (Invitrogen, Grand Island, NY) between the NdeI/XhoI restriction sites such that the in-frame fusion construct generated a His_6_-tag at the C-terminus of IFS.

Protein expression from the LFn-NADase-IFS-encoding construct, named MRW001, was insufficient for downstream studies. To improve expression, a DNA fragment encoding NADase G330D-IFS (enzymatically inactive NADase and IFS) was codon-optimized for expression in *E*. *coli* and synthesized by GENEWIZ, Inc (South Plainfield, NJ 07080). Codon-optimized *nga-ifs* was generated by OuikChange site-directed mutagenesis (Agilent Technologies) of the codon-optimized NADase G330D-IFS-encoding construct. These two constructs served as templates for PCR to generate DNA constructs encoding NADase, NADase G330D, 190NADase (aa 190–451), and 190NADase G330D using appropriate primers. Each of these PCR products was cloned between the BamHI/XhoI restriction sites in MRW001, in place of *nga-ifs* (S1 Fig).

*Generation of NADase constructs*. Codon-optimized DNA fragments encoding NADase, NADase G330D, and 190NADase were amplified by PCR using appropriate primers and cloned into the NdeI/XhoI restriction sites of pET43.1a to incorporate a C-terminal His_6_-tag on the IFS protein.

*Generation of IFS and LFn constructs*. In the first step, DNA fragments encoding an N-terminally His_6_-tagged Sumo protein, codon-optimized IFS, and LFn were amplified by PCR in separate reactions [47,48]. These PCR products served as template for overlap PCR to generate the His_6_-Sumo-IFS and His_6_-Sumo-LFn-encoding constructs, which were subsequently cloned into the NdeI/XhoI restriction sites in the pET43.1a vector.

### Expression and purification of recombinant proteins

Recombinant proteins used in this study are described in Table 1.

**Table 1:** Recombinant proteins used in this study.

Construct	Description	Reference
LFn-NADase	LFn (residues1-263 of LF) fused with NADase residues 38–451	This study
LFn-NADase G330D	LFn (residues1-263 of LF) fused with NADase residues 38–451. Residue 330 of NADase mutated from Gly to Asp	This study
LFn-190NADase	LFn (residues1-263 of LF) fused with NADase residues 190–451	This study
LFn-190NADase G330D	LFn (residues1-263 of LF) fused with NADase residues 190–451. Residue 330 of NADase mutated from Gly to Asp	This study
NADase	Full length active NADase (residues 38–451)	This study
NADase G330D	Residues 38–451 of NADase with residue 330 mutated from Gly to Asp	This study
190NADase	Residues 190–451 of NADase (“Catalytic domain”)	This study
IFS	Full length active immunity factor to NADase	This study
LFn	Residues 1–263 of LF	This study
PA	Full length functional PA	[47]
PA F427H	PA with Phe clamp mutation F427H	[49]

LFn-NADase, LFn-190NADase, NADase and their variants were expressed in BL21(DE3) cells (Invitrogen) using IPTG induction. Proteins were initially purified using Ni-charged metal affinity chromatography. Each partially purified protein preparation was loaded onto a High Performance Q column (GE) in buffer A (20 mM Tris, pH 7.5), washed with buffer A, and eluted with a gradient of 0 to 1 M NaCl in the same buffer. The proteins were then denatured in 6 M guanidinium chloride, pH 8.0, and the His-tagged IFS was removed from untagged LFn-NADase proteins by Ni-charged metal affinity chromatography. The IFS-free proteins were renatured by dialysis into buffer A containing 350 mM NaCl and 5 mM DTT. The renatured proteins were subsequently dialyzed in buffer A containing 5 mM DTT. Finally, the proteins were subjected to another round of Q column purification. Protein solutions were filter sterilized and stored at -80°C.

LFn and IFS fused with N-terminally His_6_-tagged Sumo protein were overexpressed using IPTG in BL21(DE3) cells (Invitrogen). The proteins were initially purified using Ni-charged metal affinity chromatography. Sumo was removed by cleavage with Sumo protease, and the reaction was monitored by SDS-PAGE. N-terminally His_6_-tagged Sumo and Sumo protease were removed from the now untagged protein of interest using Ni-charged metal affinity chromatography. DTT (5 mM) was added **t**o the final protein eluate for NADase constructs.

Recombinant wild type PA and PA F427H were overexpressed in the periplasm of *E*. *coli* BL21 (DE3), purified by anion-exchange chromatography, and converted to the prepore form of PA using a protocol published elsewhere [50].

### Circular dichroism (CD) spectroscopy

NADase and NADaseG330D were dialyzed against 10 mM sodium phosphate, 0.5 mM DTT, pH 8.0, and introduced at a concentration of 3.55 μM (determined by A_280_ measurements) into a stoppered 0.1 cm quartz cuvette. Equal concentration of the two proteins was confirmed by SDS-PAGE and Coomassie staining. CD spectra were measured in a JASCO J-815 Spectropolarimeter at 20°C from 185–260 nm in 0.5 nm steps with a 1 nm bandwidth. Five scans were averaged and smoothed, a background buffer-only spectrum was subtracted, and the data for the two protein species were plotted and overlayed to assess similarity.

### Differential scanning fluorimetry

Differential scanning fluorimetry was used to calculate the melting temperature of NADase, IFS, or NADase-IFS complex. A 10 μM solution of each protein was prepared in PBS containing 5X SYPRO Orange (Sigma), and the solution was dispensed in wells of a 96-well PCR plate. The plate was subjected to a temperature scan from 10 to 93°C at a rate of 1°C min^−1^ in an ABI Prism 7300 real time PCR instrument (Applied Biosystems/Invitrogen) using an excitation wavelength of 492 nm; fluorescence emission was recorded at 610 nm. Fluorescence emission of SYPRO Orange in aqueous solution increases upon binding to hydrophobic regions of proteins exposed by temperature-induced protein unfolding. The peak of the curve of the first derivative of the measured fluorescence intensity, plotted as a function of temperature, represents the melting temperature of the protein.

### Determination of NADase activity of recombinant proteins

NADase activity of the recombinant proteins was determined as described (Bricker et al., 2002). Briefly, two-fold serial dilutions of NADase, LFn-NADase, or LFn-190NADase were incubated with 0.67 mM NAD^+^ for a period of 1 h at 37°C. The reaction was then terminated by the addition of 2 M NaOH and the fluorescence of uncleaved NAD^+^ was allowed to develop for 1 h, at which point the plates were read in a fluorimeter with excitation/emission wavelengths of 355nm/560nm. Samples without NADase served as controls. The results were expressed as fraction of total NAD^+^ that was cleaved at a given NADase concentration.

### Assessment of IFS binding ability of enzymatically inactive variants of LFn-NADase constructs

Thirty-five nM NADase was added to 17.5 nM, 35 nM and 70 nM of LFn-NADase G330D, LFn-190NADase G330D and NADase G330D in a 96-well plate. Seventy nM IFS, sufficient to completely inhibit enzymatic activity of 35 nM NADase, was then added to the wells. To this mixture, 0.67 mM NAD^+^ was added and the reaction incubated for a period of 1 h at 37°C. The reaction was then terminated by the addition of 2 M NaOH and the fluorescence of uncleaved NAD^+^ was allowed to develop for 1 h at which point the plates were read in a fluorimeter with excitation/emission wavelengths of 355nm/560nm. Samples without NADase served as controls. The results were expressed as percentage inhibition of NADase activity. Complete cleavage of NAD was labeled as 0% inhibition of NADase activity and no cleavage was labeled as 100% inhibition of NADase activity.

### Measurement of intracellular NADase activity

OKP7 cells were grown in 6-well dishes at 37°C in 5% CO_2_ to approximately 70% confluence (~2x10^5^ cells/well). Cells were washed and incubated in KSFM containing GAS at a multiplicity of infection (MOI) of 10 unless otherwise indicated or supplemented with 20 nM PA and LFn-NADase at 10^−8^, 10^−9^, or 10^−10^ M for 2 h. A control lacking PA protein was also included. Fifteen min prior to harvesting cells, clindamycin (10 μg/ml) was added to prevent NADase production by GAS during sample processing. For intracellular NADase measurements, cells were washed, trypsinized, and permeabilized by incubation in PBS containing saponin (0.005% w/v) and protease inhibitors for 20 min at 37°C. Cells were removed by centrifugation for 2 min at maximum speed on a bench-top centrifuge and the supernatant containing cytosolic material was passed through a 0.2 μm filter. This filtrate, the cytosolic fraction, was kept on ice until NADase measurement. NADase activity was determined as previously described [17]. Experiments were performed three times. Intracellular activity was represented as the percentage NAD^+^ substrate depletion.

### Planar lipid bilayer experiments

Planar phospholipid bilayer experiments were performed in a Warner Instruments Planar Lipid Bilayer Workstation (BC 525D, Hamden, CT). Planar bilayers were formed by painting a 35 mM solution of 1,2-diphytanoyl-sn-glycerol-3-phosphocholine (DPhPC) in n-decane (Avanti Polar Lipids, Alabaster, AL) on a 200 μm aperture of a Delrin cup in a Lucite chamber. The aperture separated two compartments, each containing one ml of 100 mM KCl, 1 mM ethylenediaminetetraacetic acid (EDTA), and 10 mM each of sodium oxalate, potassium phosphate, and 2-(N-morpholino)ethanesulfonic acid (MES), pH 5.5. Both compartments were stirred continuously.

Upon formation of a bilayer membrane, up to 5 μg PA prepore (25 pM) was added to the *cis* compartment in the presence of a constant voltage of +20 mV with respect to the *trans* compartment. After incorporation of PA pores as monitored by conductance across the membrane, the *cis* compartment was perfused to remove any free PA. Once the current had stabilized, 1 μg of LFn-NADase or a variant was added to the *cis* compartment, and interaction with PA channels was monitored by the decrease in conductance. After occlusion of PA pores had reached a steady state, excess LFn-NADase was removed by perfusion of the *cis* chamber. KOH was then added to the *trans* compartment to raise the pH of the buffer to 7.5. An increase in conductance indicated that the pH gradient between the *cis* and *trans* compartment had triggered the translocation of LFn-NADase across the PA pore into the *trans* compartment.

### Protein synthesis inhibition assay

OKP7 cells were plated in a 96-well plate at a density of 10^4^ cells/well approximately 40 h prior to the protein synthesis inhibition assay. PA (20 nM) and LFn-NADase diluted in KSFM were added to the plates. The plates were then incubated at 37°C for 24 h, after which toxin-containing medium was removed and was replaced with L-Leucine-deficient F-12 medium supplemented with L-[4, 5-^3^H] Leucine (Perkin Elmer). The plates were incubated for 1 h at 37°C. Next, the plates were washed with ice-cold PBS, liquid scintillation cocktail was added, and incorporation of radioactivity in the cells was measured in a scintillation counter. Results were normalized and expressed as a fraction of the radioactivity incorporated in OKP7 cells that were not treated with toxin.

### Intracellular survival assay

OKP7 cells were infected at an MOI of 10 with GAS that had been grown to exponential phase (A_600nm_~0.25) and washed twice in KSFM. When appropriate, PA (20 nM) and LFn-NADase (1 nM or 0.1 nM) were added to the cells at the time of infection. Infected cell monolayers were treated with 20 μg/ml penicillin G and 200 μg/ml gentamicin for 45 minutes beginning 1 h 15 min post-infection. At 2 h post-infection, viable intracellular bacteria were quantified as described previously [51]. To determine intracellular survival at later time points, infected monolayers were washed at 2 h post-infection and fresh medium containing penicillin G (1 μg/ml), but not PA or LFn-NADase, was added. Infected monolayers were incubated for 4 h or 24 h post-infection, at which times the total intracellular CFU were determined as above.

### Confocal microscopy

OKP7 cells were cultured on coverslips in 24-well plates. Cells were infected with GAS as described above except that antibiotics were omitted to prevent cellular uptake of non-viable bacteria. Instead, extracellular bacteria were removed by extensively washing the cells with PBS at 2 h post infection, after which the cells were incubated in fresh KSFM. Infected cells were processed 1 h, 3 h, or 6 h post-infection. At each of these time points, monolayers were washed three times with PBS and extracellular GAS were stained with Alexa Fluor 660-conjugated anti-GAS IgG at 4°C for 15 min in the dark. Excess unbound antibody was removed by washing with PBS. Subsequently, cells were fixed and permeabilized by incubation in ice-cold methanol at −20°C for 5 min. Cells were then washed three times with PBS and incubated at room temperature for 1 h with mouse anti-LAMP-1 IgG. After three washes in PBS, cells were incubated for 1 h with goat anti-mouse Alexa Fluor 568-conjugated IgG and with Alexa Fluor 488-conjugated anti-GAS IgG at room temperature in the dark for 1 h. Slides were mounted using Prolong Gold (Molecular Probes) and stored at room temperature in the dark for 16–24 h prior to imaging. Confocal microscopy was performed at the Harvard Digestive Diseases Center core facility as previously described [51]. Images were acquired and analyzed using Slidebook 5 and Slidebook 6 (Intelligent Imaging Innovations, Denver, CO). For quantification, co-localization of intracellular bacteria with LAMP-1 marker was determined from three independent experiments. Images were evaluated by an observer who was blind to the experimental conditions. At least 100 intracellular bacteria were scored for each experiment.

### Cytotoxicity assays

OKP7 cells were cultured on coverslips in 24-well plates and grown to 40–50% confluence. Cells were then incubated with medium containing 20 nM PA and either 100 nM LFn-NADase or 100 nM LFn-NADase G330D for a period of 48 h. Cells that were not treated with any toxin served as a negative control. Cells were then washed with PBS and incubated with 500 μl of PBS containing 1 μg/ml propidium iodide for a period of 30 min at room temperature in the dark. Cells were then visualized under a Nikon Eclipse TS100 fluorescence microscope with a standard TRITC filter set (Ex 535/50, Em 610/75, DM 565) and images were acquired. For easy visualization, images showing dead cells stained with propidium iodide were colored red and merged with bright-field images showing the total number of cells (ImageJ software).

### Statistical analysis

Significance of differences between experimental groups was assessed by Student’s t-test. P values of less than 0.05 were considered statistically significant.

## Supporting Information

S1 Fig Schematic diagrams of expression constructs for recombinant proteins prepared in this study. (PDF)

S2 Fig Evidence that enzymatically inactive variants of NADase retain their native conformations after purification. (A) Ability of enzymatically inactive variants of LFn-NADase to compete for binding of NADase to IFS. Plots demonstrate dose-dependent inhibition of NADase activity. Reactions contained 35 nM NADase, 70 nM IFS, and the indicated concentration of inactive variant protein. (B) Circular dichroism spectra of NADase and NADaseG330D after purification and renaturation. The nearly identical spectra suggest similar secondary structures.
(PDF)

S3 Fig Increase in NADase melting temperature associated with binding of IFS. Plots represent melting curves of NADase, IFS, and NADase-IFS complex as determined by differential scanning fluorimetry. The peak of the curve of the first derivative of the measured fluorescence intensity, plotted as a function of temperature, represents the melting temperature of the protein.
(PDF)

S4 Fig Dependence on PA of LFn-NADase translocation into OKP7 keratinocytes. Data represent inhibition of protein synthesis by PA-mediated translocation of LFn-NADase in OKP7 cells. Protein synthesis was assayed by incorporation of tritiated leucine and is plotted as the fraction of activity in OKP7 cells in the absence of recombinant protein. Inhibition of protein synthesis by LFn-NADase or LFn-190NADase did not occur in the absence of PA, which is required for LFn-mediated translocation.
(PDF)

S5 Fig Intracellular delivery of NADase varies with multiplicity of infection. NADase activity was measured in the cytosolic fraction of OKP7 cell lysates after 2 hours exposure of OKP7 cells to GAS strain 188 at the indicated multiplicity of infection. Values represent mean±SEM from three independent experiments.
(PDF)

## References

[1] Aziz RK, Kotb M (2008) Rise and persistence of global M1T1 clone of Streptococcus pyogenes. Emerging Infect Dis 14: 1511–1517. 10.3201/eid1410.071660 18826812 PMC2609857

[2] Cleary PP, Kaplan EL, Handley JP, Wlazlo A, Kim MH, et al (1992) Clonal basis for resurgence of serious *Streptococcus pyogenes* disease in the 1980s. Lancet 339: 518–521. 1346879 10.1016/0140-6736(92)90339-5

[3] Musser JM, Kapur V, Szeto JXP, Swanson D, Martin D (1995) Genetic diversity and relationships among *Streptococcus pyogenes* strains expressing serotype M1 protein: recent intercontinental spread of a subclone causing episodes of invasive disease. Infect Immun 63: 994–1003. 7868273 10.1128/iai.63.3.994-1003.1995 PMC173101

[4] Stevens DL, Salmi DB, McIndoo ER, Bryant AE (2000) Molecular epidemiology of nga and NAD glycohydrolase/ADP-ribosyltransferase activity among *Streptococcus pyogenes* causing streptococcal toxic shock syndrome. J Infect Dis 182: 1117–1128. 10979908 10.1086/315850

[5] Sumby P, Porcella SF, Madrigal AG, Barbian KD, Virtaneva K, et al (2005) Evolutionary origin and emergence of a highly successful clone of serotype M1 group a Streptococcus involved multiple horizontal gene transfer events. J Infect Dis 192: 771–782. 16088826 10.1086/432514

[6] Tatsuno I, Sawai J, Okamoto A, Matsumoto M, Minami M, et al (2007) Characterization of the NAD-glycohydrolase in streptococcal strains. Microbiology 153: 4253–4260. 18048938 10.1099/mic.0.2007/009555-0

[7] Ajdic D, McShan WM, Savic DJ, Gerlach D, Ferretti JJ (2000) The NAD-glycohydrolase (nga) gene of *Streptococcus pyogenes* . FEMS Microbiol Lett 191: 235–241. 11024269 10.1111/j.1574-6968.2000.tb09345.x

[8] Kimoto H, Fujii Y, Hirano S, Yokota Y, Taketo A (2006) Genetic and biochemical properties of streptococcal NAD-glycohydrolase inhibitor. J Biol Chem 281: 9181–9189. 16380378 10.1074/jbc.M506879200

[9] Meehl MA, Pinkner JS, Anderson PJ, Hultgren SJ, Caparon MG (2005) A Novel Endogenous Inhibitor of the Secreted Streptococcal NAD-Glycohydrolase. PLoS Pathog 1: e35 16333395 10.1371/journal.ppat.0010035 PMC1298937

[10] Maamary PG, Ben Zakour NL, Cole JN, Hollands A, Aziz RK, et al (2012) Tracing the evolutionary history of the pandemic group A streptococcal M1T1 clone. FASEB J 26: 4675–4684. 10.1096/fj.12-212142 22878963 PMC3475248

[11] Nasser W, Beres SB, Olsen RJ, Dean MA, Rice KA, et al (2014) Evolutionary pathway to increased virulence and epidemic group A Streptococcus disease derived from 3,615 genome sequences. Proc Natl Acad Sci U S A 111: E1768–1776. 10.1073/pnas.1403138111 24733896 PMC4035937

[12] Sumby P, Barbian KD, Gardner DJ, Whitney AR, Welty DM, et al (2005) Extracellular deoxyribonuclease made by group A Streptococcus assists pathogenesis by enhancing evasion of the innate immune response. Proc Natl Acad Sci U S A 102: 1679–1684. 15668390 10.1073/pnas.0406641102 PMC547841

[13] Carlson AS, Kellner A, Bernheimer AW, Freeman EB (1957) A streptococcal enzyme that acts specifically upon diphosphopyridine nucleotide: characterization of the enzyme and its separation from streptolysin O. J Exp Med 106: 15–26. 13439111 10.1084/jem.106.1.15 PMC2136732

[14] Fehrenbach FJ (1971) Identity of streptolysin-O and NAD-glycohydrolase (EC 3.2.2.5). Z Naturforsch B 26: 1336–1338. 4401888 10.1515/znb-1971-1224

[15] Shany S, Grushoff PS, Bernheimer AW (1973) Physical separation of streptococcal nicotinamide adenine dinucleotide glycohydrolase from streptolysin O. Infect Immun 7: 731–734. 4357989 10.1128/iai.7.5.731-734.1973 PMC422752

[16] Madden JC, Ruiz N, Caparon M (2001) Cytolysin-mediated translocation (CMT): a functional equivalent of type III secretion in gram-positive bacteria. Cell 104: 143–152. 11163247 10.1016/s0092-8674(01)00198-2

[17] Bricker AL, Cywes C, Ashbaugh CD, Wessels MR (2002) NAD+-glycohydrolase acts as an intracellular toxin to enhance the extracellular survival of group A streptococci. Mol Microbiol 44: 257–269. 11967084 10.1046/j.1365-2958.2002.02876.x

[18] Bricker AL, Carey VJ, Wessels MR (2005) Role of NADase in Virulence in Experimental Invasive Group A Streptococcal Infection. Infect Immun 73: 6562–6566. 16177331 10.1128/IAI.73.10.6562-6566.2005 PMC1230891

[19] Tatsuno I, Isaka M, Minami M, Hasegawa T (2010) NADase as a target molecule of in vivo suppression of the toxicity in the invasive M-1 group A Streptococcal isolates. BMC microbiology 10: 144 10.1186/1471-2180-10-144 20470439 PMC2887803

[20] Michos A, Gryllos I, Hakansson A, Srivastava A, Kokkotou E, et al (2006) Enhancement of streptolysin O activity and intrinsic cytotoxic effects of the group A streptococcal toxin, NAD-glycohydrolase. J Biol Chem 281: 8216–8223. 16431917 10.1074/jbc.M511674200

[21] O'Seaghdha M, Wessels MR (2013) Streptolysin O and its Co-Toxin NAD-glycohydrolase Protect Group A Streptococcus from Xenophagic Killing. PLoS pathog 9: e1003394 10.1371/journal.ppat.1003394 23762025 PMC3675196

[22] Feld GK, Thoren KL, Kintzer AF, Sterling HJ, Tang II, et al (2010) Structural basis for the unfolding of anthrax lethal factor by protective antigen oligomers. Nat Struct Mol Biol 17: 1383–1390. 10.1038/nsmb.1923 21037566 PMC3133606

[23] Kintzer AF, Thoren KL, Sterling HJ, Dong KC, Feld GK, et al (2009) The protective antigen component of anthrax toxin forms functional octameric complexes. J Mol Biol 392: 614–629. 10.1016/j.jmb.2009.07.037 19627991 PMC2742380

[24] Young JA, Collier RJ (2007) Anthrax toxin: receptor binding, internalization, pore formation, and translocation. Annual review of biochemistry 76: 243–265. 17335404 10.1146/annurev.biochem.75.103004.142728

[25] Abrami L, Liu S, Cosson P, Leppla SH, van der Goot FG (2003) Anthrax toxin triggers endocytosis of its receptor via a lipid raft-mediated clathrin-dependent process. J Cell Biol 160: 321–328. 12551953 10.1083/jcb.200211018 PMC2172673

[26] Boll W, Ehrlich M, Collier RJ, Kirchhausen T (2004) Effects of dynamin inactivation on pathways of anthrax toxin uptake. Eur J Cell Biol 83: 281–288. 15511085 10.1078/0171-9335-00373

[27] Blaustein RO, Koehler TM, Collier RJ, Finkelstein A (1989) Anthrax toxin: channel-forming activity of protective antigen in planar phospholipid bilayers. Proc Natl Acad Sci U S A 86: 2209–2213. 2467303 10.1073/pnas.86.7.2209 PMC286881

[28] Koehler TM, Collier RJ (1991) Anthrax toxin protective antigen: low-pH-induced hydrophobicity and channel formation in liposomes. Mol Microbiol 5: 1501–1506. 1787799 10.1111/j.1365-2958.1991.tb00796.x

[29] Wesche J, Elliott JL, Falnes PO, Olsnes S, Collier RJ (1998) Characterization of membrane translocation by anthrax protective antigen. Biochemistry 37: 15737–15746. 9843379 10.1021/bi981436i

[30] Quinn CP, Singh Y, Klimpel KR, Leppla SH (1991) Functional mapping of anthrax toxin lethal factor by in-frame insertion mutagenesis. J Biol Chem 266: 20124–20130. 1939073

[31] Arora N, Leppla SH (1993) Residues 1–254 of anthrax toxin lethal factor are sufficient to cause cellular uptake of fused polypeptides. J Biol Chem 268: 3334–3341. 8429009

[32] Arora N, Leppla SH (1994) Fusions of anthrax toxin lethal factor with shiga toxin and diphtheria toxin enzymatic domains are toxic to mammalian cells. Infect Immun 62: 4955–4961. 7927776 10.1128/iai.62.11.4955-4961.1994 PMC303212

[33] Ballard JD, Collier RJ, Starnbach MN (1996) Anthrax toxin-mediated delivery of a cytotoxic T-cell epitope in vivo. Proc Natl Acad Sci U S A 93: 12531–12534. 8901616 10.1073/pnas.93.22.12531 PMC38026

[34] Goletz TJ, Klimpel KR, Arora N, Leppla SH, Keith JM, et al (1997) Targeting HIV proteins to the major histocompatibility complex class I processing pathway with a novel gp120-anthrax toxin fusion protein. Proc Natl Acad Sci U S A 94: 12059–12064. 9342362 10.1073/pnas.94.22.12059 PMC23701

[35] Milne JC, Blanke SR, Hanna PC, Collier RJ (1995) Protective antigen-binding domain of anthrax lethal factor mediates translocation of a heterologous protein fused to its amino- or carboxy-terminus. Mol Microbiol 15: 661–666. 7783638 10.1111/j.1365-2958.1995.tb02375.x

[36] Ghosh J, Anderson PJ, Chandrasekaran S, Caparon MG (2010) Characterization of Streptococcus pyogenes beta-NAD+ glycohydrolase: re-evaluation of enzymatic properties associated with pathogenesis. J Biol Chem 285: 5683–5694. 10.1074/jbc.M109.070300 20018886 PMC2820796

[37] Ghosh J, Caparon MG (2006) Specificity of Streptococcus pyogenes NAD(+) glycohydrolase in cytolysin-mediated translocation. Mol Microbiol 62: 1203–1214. 17042787 10.1111/j.1365-2958.2006.05430.x

[38] Chandrasekaran S, Ghosh J, Port GC, Koh EI, Caparon MG (2013) Analysis of polymorphic residues reveals distinct enzymatic and cytotoxic activities of the Streptococcus pyogenes NAD+ glycohydrolase. J Biol Chem 288: 20064–20075. 10.1074/jbc.M113.481556 23689507 PMC3707703

[39] Riddle DJ, Bessen DE, Caparon MG (2010) Variation in Streptococcus pyogenes NAD+ glycohydrolase is associated with tissue tropism. J Bacteriol 192: 3735–3746. 10.1128/JB.00234-10 20494994 PMC2897333

[40] Krantz BA, Melnyk RA, Zhang S, Juris SJ, Lacy DB, et al (2005) A phenylalanine clamp catalyzes protein translocation through the anthrax toxin pore. Science 309: 777–781. 16051798 10.1126/science.1113380 PMC1815389

[41] Hakansson A, Bentley CC, Shakhnovic EA, Wessels MR (2005) Cytolysin-dependent evasion of lysosomal killing. Proc Natl Acad Sci U S A 102: 5192–5197. 15795386 10.1073/pnas.0408721102 PMC555683

[42] Nakagawa I, Amano A, Mizushima N, Yamamoto A, Yamaguchi H, et al (2004) Autophagy defends cells against invading group A Streptococcus. Science 306: 1037–1040. 15528445 10.1126/science.1103966

[43] Crowe DL, Hu L, Gudas LJ, Rheinwald JG (1991) Variable expression of retinoic acid receptor (RARß) mRNA in human oral and epidermal keratinocytes; relation to keratin 19 expression and keratinization potential. Differentiation 48: 199–208. 1725165 10.1111/j.1432-0436.1991.tb00258.x

[44] Dickson MA, Hahn WC, Ino Y, Ronfard V, Wu JY, et al (2000) Human keratinocytes that express hTERT and also bypass a p16INK4a-enforced mechanism that limits life span become immortal yet retain normal growth and differentiation characteristics. Mol Cell Biol 20: 1436–1447. 10648628 10.1128/mcb.20.4.1436-1447.2000 PMC85304

[45] Ashbaugh CD, Warren HB, Carey VJ, Wessels MR (1998) Molecular analysis of the role of the group A streptococcal cysteine protease, hyaluronic acid capsule, and M protein in a murine model of human invasive soft-tissue infection. J Clin Invest 102: 550–560. 9691092 10.1172/JCI3065 PMC508916

[46] Hill JE, Wannamaker LW (1981) Identification of a lysin associated with a bacteriophage (A25) virulent for group A streptococci. J Bacteriol 145: 696–703. 7007344 10.1128/jb.145.2.696-703.1981 PMC217168

[47] Christensen KA, Krantz BA, Melnyk RA, Collier RJ (2005) Interaction of the 20 kDa and 63 kDa fragments of anthrax protective antigen: kinetics and thermodynamics. Biochemistry 44: 1047–1053. 15654761 10.1021/bi047791s

[48] Sharma O, Collier RJ (2014) Polylysine-Mediated Translocation of the Diphtheria Toxin Catalytic Domain through the Anthrax Protective Antigen Pore. Biochemistry 53: 6934–6940. 10.1021/bi500985v 25317832 PMC4230326

[49] Sun J, Lang AE, Aktories K, Collier RJ (2008) Phenylalanine-427 of anthrax protective antigen functions in both pore formation and protein translocation. Proc Natl Acad Sci U S A 105: 4346–4351. 10.1073/pnas.0800701105 18334631 PMC2393744

[50] Miller CJ, Elliott JL, Collier RJ (1999) Anthrax protective antigen: prepore-to-pore conversion. Biochemistry 38: 10432–10441. 10441138 10.1021/bi990792d

[51] Logsdon LK, Hakansson AP, Cortes G, Wessels MR (2011) Streptolysin O inhibits clathrin-dependent internalization of group A Streptococcus. mBio 2: e00332–00310. 10.1128/mBio.00332-10 21325037 PMC3039441

