# Supplementary material for: NAD+-Glycohydrolase Promotes Intracellular Survival of Group A Streptococcus
Source: PLoS Pathog. 2016 Mar 3;12(3):e1005468. doi: 10.1371/journal.ppat.1005468 (PMC4777570; doi:10.1371/journal.ppat.1005468)
Supplement: S1 Fig — (PDF) [file ppat.1005468.s001.pdf]

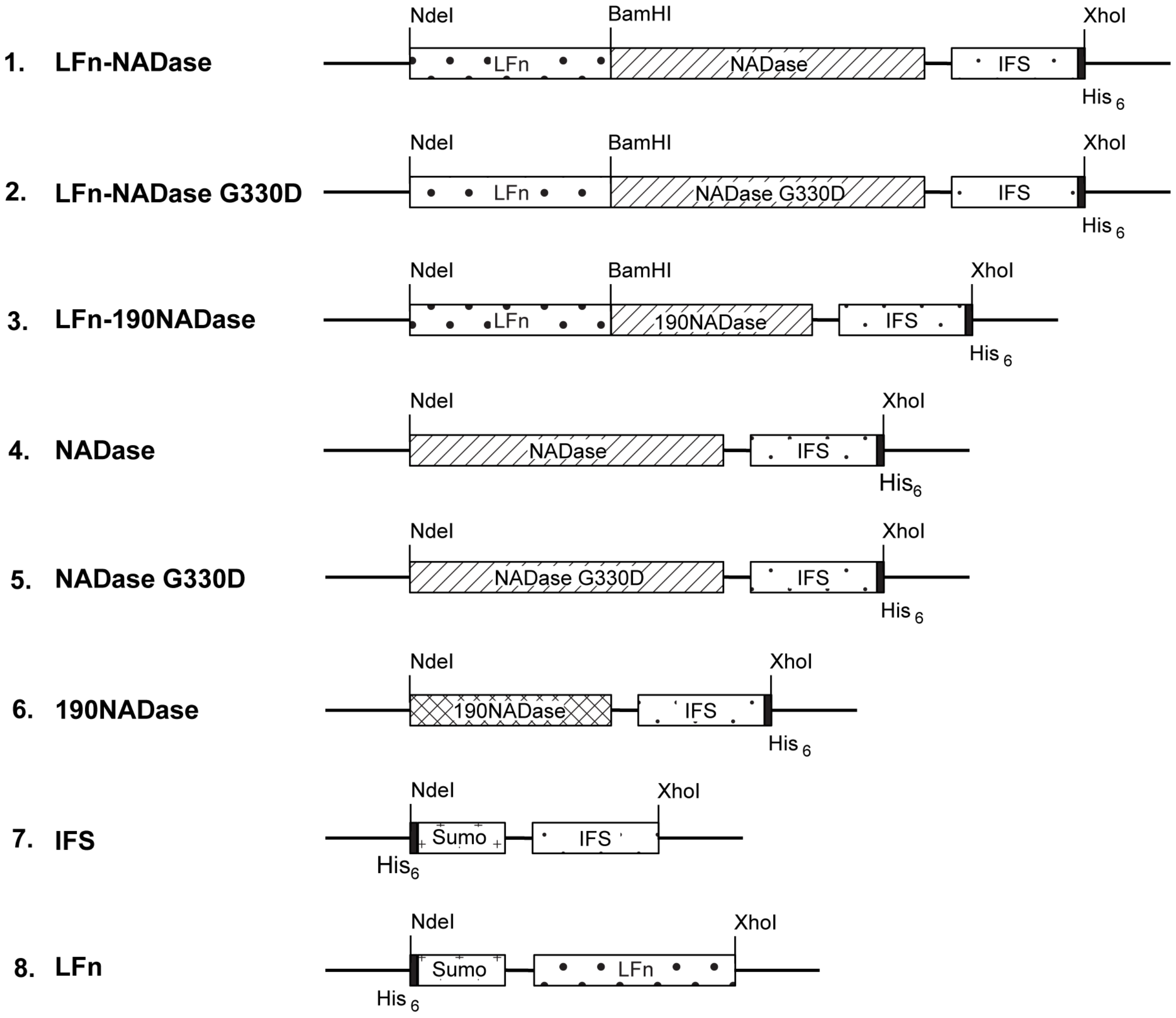

S1 Fig. Schematic diagrams of expression constructs for recombinant proteins prepared in this study.
