# Supplementary material for: NAD+-Glycohydrolase Promotes Intracellular Survival of Group A Streptococcus
Source: PLoS Pathog. 2016 Mar 3;12(3):e1005468. doi: 10.1371/journal.ppat.1005468 (PMC4777570; doi:10.1371/journal.ppat.1005468)
Supplement: S2 Fig — (A) Ability of enzymatically inactive variants of LFn-NADase to compete for binding of NADase to IFS. Plots demonstrate dose-dependent inhibition of NADase activity. Reactions contained 35 nM NADase, 70 nM IFS, and the indicated concentration of inactive variant protein. (B) Circular dichroism spectra of NADase and NADaseG330D after purification and renaturation. The nearly identical spectra suggest similar secondary structures. (PDF) [file ppat.1005468.s002.pdf]

A.

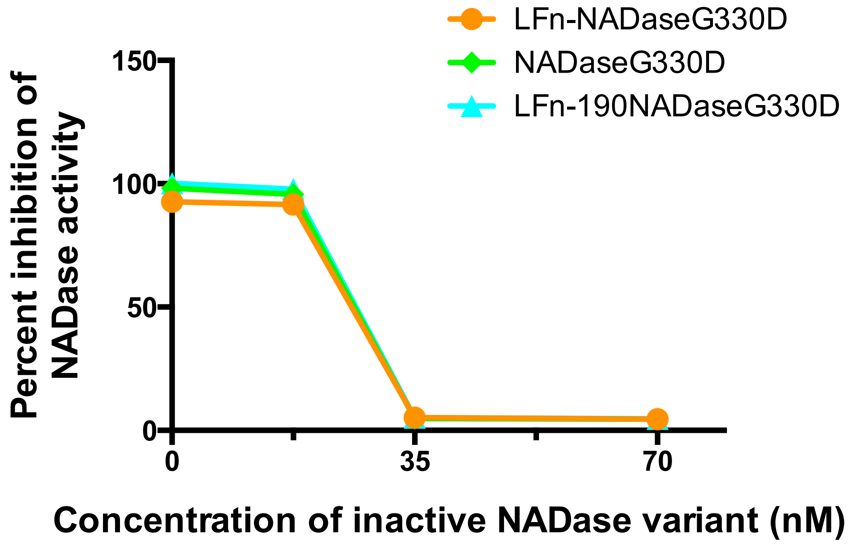

B.

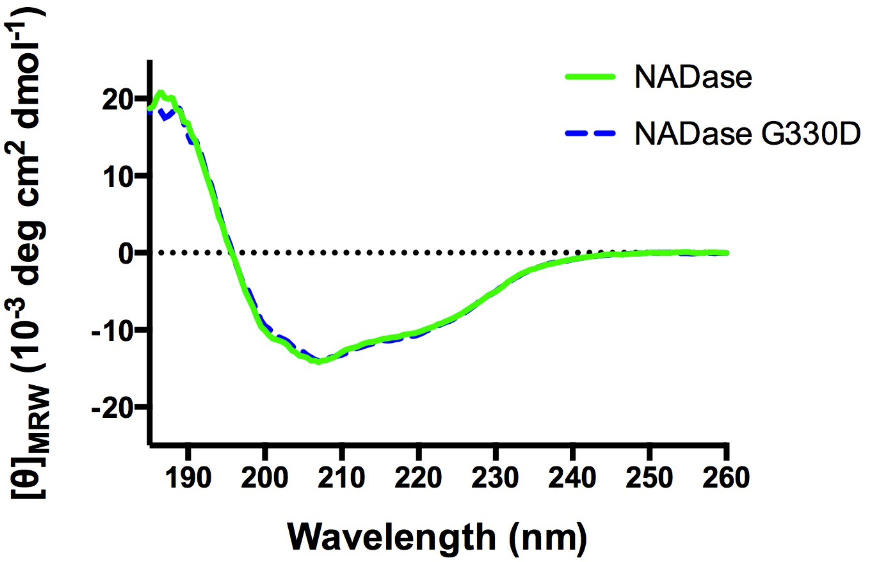

S2 Fig. Evidence that enzymatically inactive variants of NADase retain their native conformations after purification. (A) Ability of enzymatically inactive variants of LFn-NADase to compete for binding of NADase to IFS. Plots demonstrate dose-dependent inhibition of NADase activity. Reactions contained 35 nM NADase, 70 nM IFS, and the indicated concentration of inactive variant protein. (B) Circular dichroism spectra of NADase and NADaseG330D after purification and renaturation. The nearly identical spectra suggest similar secondary structures.
