# Supplementary material for: NAD+-Glycohydrolase Promotes Intracellular Survival of Group A Streptococcus
Source: PLoS Pathog. 2016 Mar 3;12(3):e1005468. doi: 10.1371/journal.ppat.1005468 (PMC4777570; doi:10.1371/journal.ppat.1005468)
Supplement: S3 Fig — Plots represent melting curves of NADase, IFS, and NADase-IFS complex as determined by differential scanning fluorimetry. The peak of the curve of the first derivative of the measured fluorescence intensity, plotted as a function of temperature, represents the melting temperature of the protein. (PDF) [file ppat.1005468.s003.pdf]

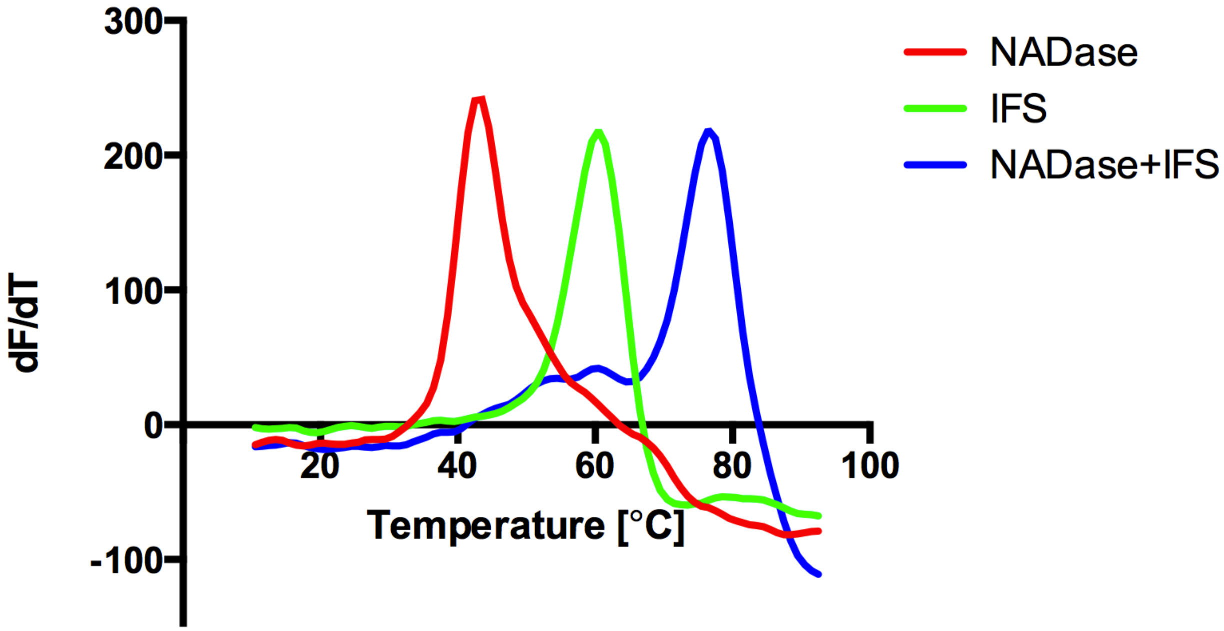

S3 Fig. Increase in NADase melting temperature associated with binding of IFS. Plots represent melting curves of NADase, IFS, and NADase-IFS complex as determined by differential scanning fluorimetry. The peak of the curve of the first derivative of the measured fluorescence intensity, plotted as a function of temperature, represents the melting temperature of the protein.
