# Supplementary material for: NAD+-Glycohydrolase Promotes Intracellular Survival of Group A Streptococcus
Source: PLoS Pathog. 2016 Mar 3;12(3):e1005468. doi: 10.1371/journal.ppat.1005468 (PMC4777570; doi:10.1371/journal.ppat.1005468)
Supplement: S4 Fig — Data represent inhibition of protein synthesis by PA-mediated translocation of LFn-NADase in OKP7 cells. Protein synthesis was assayed by incorporation of tritiated leucine and is plotted as the fraction of activity in OKP7 cells in the absence of recombinant protein. Inhibition of protein synthesis by LFn-NADase or LFn-190NADase did not occur in the absence of PA, which is required for LFn-mediated translocation. (PDF) [file ppat.1005468.s004.pdf]

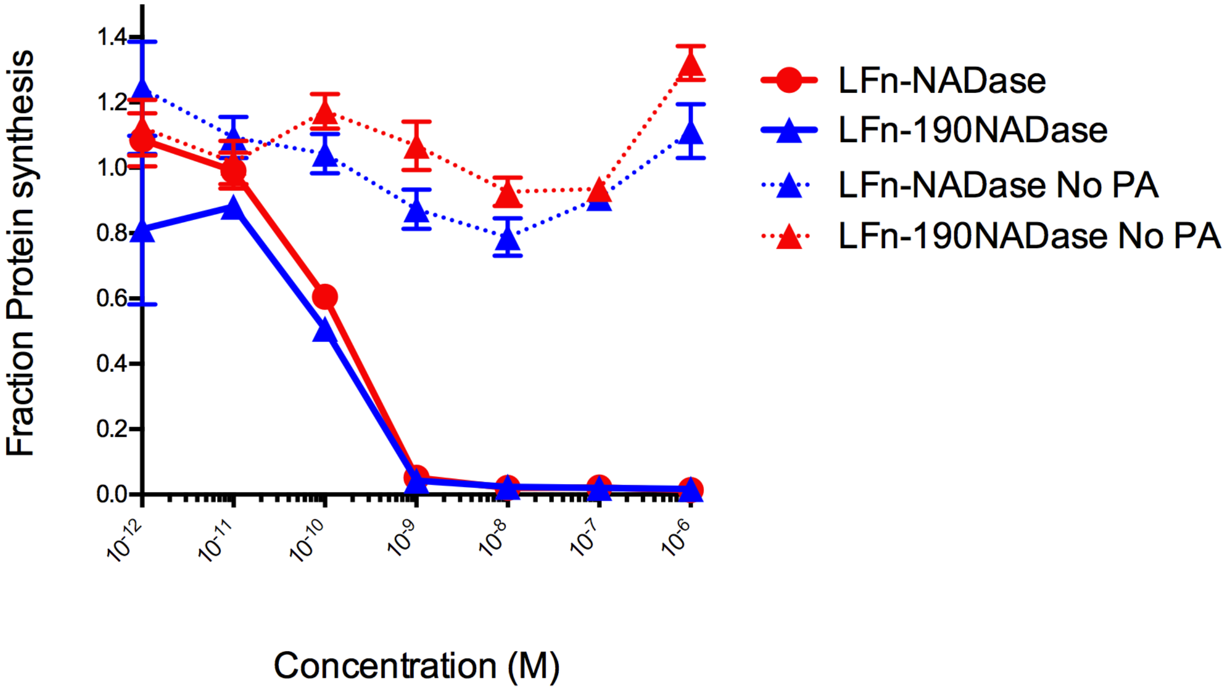

S4 Fig. Dependence on PA of LFn-NADase translocation into OKP7 keratinocytes. Data represent inhibition of protein synthesis by PA-mediated translocation of LFn-NADase in OKP7 cells. Protein synthesis was assayed by incorporation of tritiated leucine and is plotted as the fraction of activity in OKP7 cells in the absence of recombinant protein. Inhibition of protein synthesis by LFn-NADase or LFn-190NADase did not occur in the absence of PA, which is required for LFn-mediated translocation.
