# Supplementary material for: NAD+-Glycohydrolase Promotes Intracellular Survival of Group A Streptococcus
Source: PLoS Pathog. 2016 Mar 3;12(3):e1005468. doi: 10.1371/journal.ppat.1005468 (PMC4777570; doi:10.1371/journal.ppat.1005468)
Supplement: S5 Fig — NADase activity was measured in the cytosolic fraction of OKP7 cell lysates after 2 hours exposure of OKP7 cells to GAS strain 188 at the indicated multiplicity of infection. Values represent mean±SEM from three independent experiments. (PDF) [file ppat.1005468.s005.pdf]

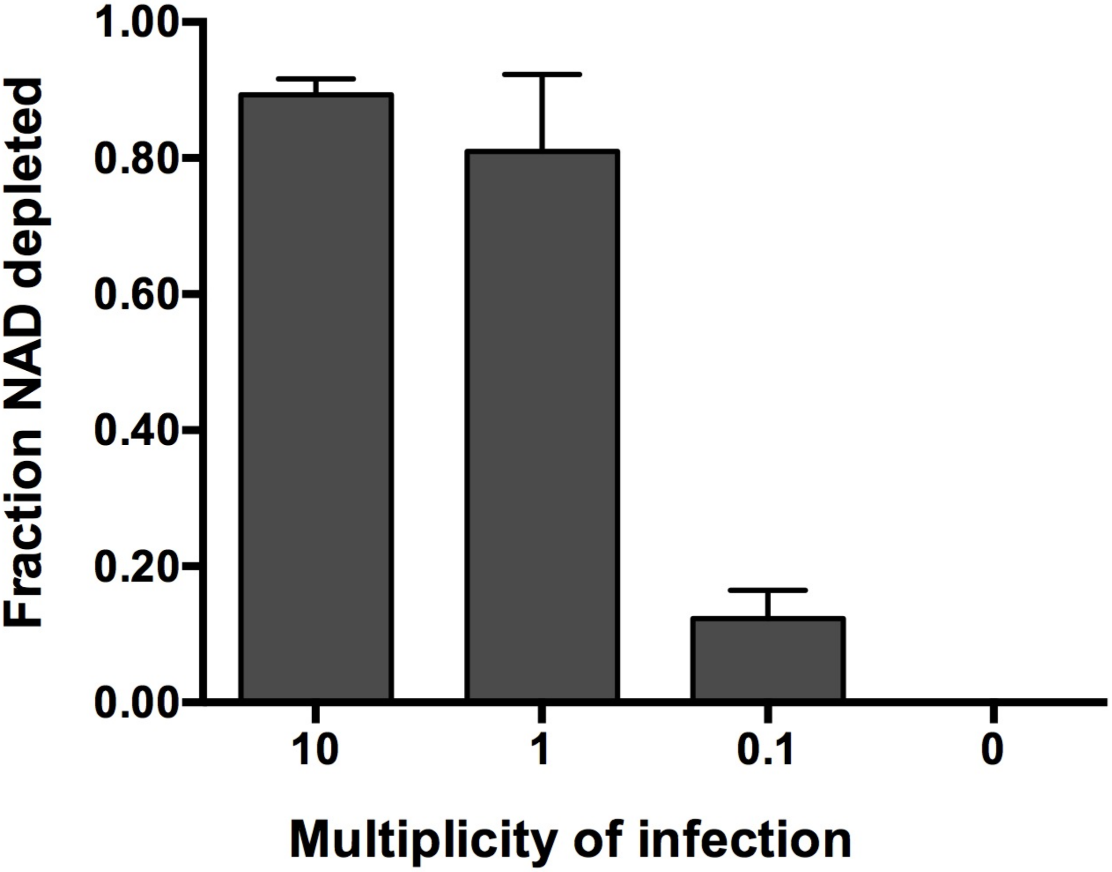

S5 Fig. Intracellular delivery of NADase varies with multiplicity of infection. NADase activity was measured in the cytosolic fraction of OKP7 cell lysates after 2 hours exposure of OKP7 cells to GAS strain 188 at the indicated multiplicity of infection. Values represent mean±SEM from three independent experiments.
